# Gut Dysbiosis and Its Associations with Gut Microbiota-Derived Metabolites in Dogs with Myxomatous Mitral Valve Disease

**DOI:** 10.1128/mSystems.00111-21

**Published:** 2021-04-20

**Authors:** Qinghong Li, Éva Larouche-Lebel, Kerry A. Loughran, Terry P. Huh, Jan S. Suchodolski, Mark A. Oyama

**Affiliations:** a Nestlé Purina Research, St. Louis, Missouri, USA; b Department of Clinical Sciences and Advanced Medicine, School of Veterinary Medicine, University of Pennsylvania, Philadelphia, Pennsylvania, USA; c Gastrointestinal Laboratory, Department of Small Animal Clinical Sciences, College of Veterinary Medicine and Biomedical Sciences, Texas A&M University, College Station, Texas, USA; North Carolina A&T State University

**Keywords:** microbiota, trimethylamine *N*-oxide, mitral valve disease, canine, dysbiosis, bile acid, microbial metabolite, congestive heart failure, *Clostridium hiranonis*, metabolite, microbiome

## Abstract

Gut dysbiosis and gut microbiota-derived metabolites, including bile acid (BA), short-chain fatty acid, and trimethylamine *N*-oxide (TMAO), are associated with cardiovascular disease. Canine myxomatous mitral valve disease (MMVD) is a model for human MMVD. The aim of the study is to evaluate gut microbial dysbiosis and its relationship with gut-produced metabolites in dogs with MMVD. Fecal samples from 92 privately owned dogs, including 17 healthy, 23 and 27 asymptomatic MMVD dogs without (stage B1) and with (stage B2) secondary cardiac enlargement, respectively, and 25 MMVD dogs with history of congestive heart failure (stage C or D), were analyzed by 16S rRNA sequencing. Alpha and beta diversities were different between healthy and MMVD dogs (adjusted *P* < 0.05). The average dysbiosis indexes were −1.48, −0.6, 0.01, and 1.47 for healthy, B1, B2, and C/D dogs, respectively (*P* = 0.07). Dysbiosis index was negatively correlated with Clostridium hiranonis (*P* < 0.0001, *r* = −0.79). Escherichia coli, capable of trimethylamine production in the gut, had an increased abundance (adjusted *P* < 0.05) and may be responsible for the increased circulating TMAO levels in stage B2 and C/D MMVD dogs. Primary and secondary BAs showed opposite associations with *C. hiranonis*, a key BA converter (*P* < 0.0001 for both, *r* = −0.94 and 0.95, respectively). Secondary BAs appeared to promote the growth of *Fusobacterium* and *Faecalibacterium* but inhibit that of E. coli. Multivariate analysis revealed significant but weak associations between gut microbiota and several circulating metabolites, including short-chain acylcarnitines and TMAO.

**IMPORTANCE** Our study expands the current “gut hypothesis” to include gut dysbiosis at the preclinical stage, prior to the onset of heart failure. Gut dysbiosis index increases in proportion to the severity of myxomatous mitral valve disease (MMVD) and is inversely associated with Clostridium hiranonis, a key bile acid (BA) converter in the gut. Secondary BAs appear to promote the growth of beneficial bacteria but inhibit that of harmful ones. An intricate interplay between gut microbiota, gut microbiota-produced metabolites, and MMVD pathophysiological progression is implicated.

## INTRODUCTION

Canine myxomatous mitral valve disease (MMVD) is a common naturally occurring heart condition that is characterized by progressive myxomatous degeneration of the mitral valve (1, 2). Dogs with MMVD typically experience a lengthy preclinical period before progressing to clinical stage to reveal overt clinical signs of congestive heart failure (CHF). According to the consensus guideline of the American College of Veterinary Internal Medicine (ACVIM), dogs at the preclinical stage with asymptomatic MMVD and no or mild cardiac enlargement are identified as stage B1, while those with advanced cardiac remodeling and severe mitral regurgitation are classified as stage B2. Dogs with MMVD and clinical signs of CHF are classified as stage C or D (3). Canine MMVD is considered a model for human MMVD in that they share many similarities at both molecular and pathophysiological levels (4, 5). Factors that contribute to the MMVD pathogenesis and progression remain unclear.

Recently, two groups independently reported higher circulating concentrations of trimethylamine *N*-oxide (TMAO) and its nutrient precursors in dogs with MMVD and CHF than in preclinical or healthy dogs (6, 7). Increased concentrations of TMAO and its precursors in circulation were also observed in preclinical dogs versus that in healthy dogs (7). However, it is unclear whether these increases were the causes or consequences of MMVD or CHF in dogs.

Since the first study that linked cardiovascular disease with TMAO and its nutrient precursors (8), multiple gut microbiota-dependent pathways, including the trimethylamine (TMA)/TMAO pathway, short-chain fatty acid (SCFA) pathway, and primary and secondary bile acid (BA) pathways have been implicated in the development and progression of cardiovascular diseases (9–11). The gut microbiota metabolizes dietary nutrients such as l-carnitine, phosphatidylcholine, and choline to produce TMA, which enters host circulation where further metabolism by hepatic enzymes produces TMAO (8, 9, 12). The “leaky gut” hypothesis of heart failure postulates that intestinal wall edema and impaired intestinal barrier function leads to translocation of gut bacterial metabolites into the host bloodstream and resultant heightened systemic inflammation (9). The observation of elevated circulating TMAO concentration in dogs with asymptomatic MMVD suggests that the “leaky gut” begins before the onset of CHF (7). Another group of metabolites that have received considerable attention in cardiovascular disease is BAs, which facilitate intestinal absorption of dietary fat and other fat-soluble molecules. The primary BAs, such as cholic acid (CA) and chenodeoxycholic acid (CDCA), are synthesized from cholesterol in the liver (13). The majority of primary BAs are reabsorbed in the ileum by active transport, while a small amount is recycled in the upper intestine via passive diffusion (14). The remaining unrecycled primary BAs are further metabolized to produce secondary BAs, including deoxycholic acid (DCA), lithocholic acid (LCA), and ursodeoxycholic acid (UDCA) by gut microbiota in the colon (15, 16). This pool of chemically diversified BAs regulates energy metabolism and other physiological processes through multiple signaling pathways mediated by the BA-responsive receptors, including G protein-coupled membrane receptor 5 (TGR5) and farnesoid nuclear receptor (FXR) (13, 17). In addition, gut microbiota can produce large quantities of SCFAs through anaerobic fermentation. A significant portion of gut-derived SCFAs are absorbed into circulation to regulate various biological processes through signaling pathways mediated by G protein-coupled receptors. Recently, SCFAs have been shown to modulate blood pressure and prevent the development of heart failure in rodent models (18, 19). While much progress has been made toward understanding the roles of gut microbial dysbiosis on heart disease in humans and rodent models, little research has been done in dogs.

A recent study compared gut microbiota changes between 15 healthy control dogs and 35 dogs with CHF and revealed changes in microbial composition (20). In this study, we investigated the relationship among gut microbial dysbiosis and circulating metabolites as well as gut-derived metabolites such as BAs and SCFAs in healthy dogs, dogs with preclinical MMVD, and dogs with CHF secondary to MMVD. We tested our hypotheses that changes in gut microbiota occurred early during MMVD progression and that these changes are associated with gut microbiota-dependent metabolites.

## RESULTS

The 16S rRNA gene sequencing was performed on the fecal genomic DNA from 92 privately owned dogs (Table 1). Among them were 17 healthy dogs (group A), 23 stage B1 and 27 stage B2 dogs with asymptomatic MMVD (groups B1 and B2, respectively), and 25 dogs with MMVD and history of CHF (group C/D). No age difference was found except between group A and group C/D (*P* < 0.05). No differences in body weight, body condition score (BCS), or sex was observed. A total of 12.6 million paired-end sequences were obtained. The median sequence length after trimming and filtering was 443 nucleotides, with the interquartile range (IQR) between 440 and 461 nucleotides.

**TABLE 1:** Physical characteristics, echocardiography, and common cardiac medications of the dogs

Characteristic	ACVIM stage:^*a*^				*P* value^*b*^
	A	B1	B2	C/D	
Sample size (*N* = 92)	17	23	27	25	
Sex (male/female)	8/9	17/6	16/11	15/10	0.39
Age (yrs)	8.9 ± 0.5	10.3 ± 0.4	10.2 ± 0.4	11.5 ± 0.3	<0.001
Body wt (kg)	10.4 ± 1.0	9.2 ± 0.8	8.1 ± 0.6	7.8 ± 0.8	0.14
BCS (1–9)^*c*^	5.1 ± 0.1	5.5 ± 0.2	5.6 ± 0.2	5.1 ± 0.2	0.28
Cardiac medications (*n*)^*d*^					
Pimobendan	0	4	19	23	
Furosemide (Lasix)	0	1	2	21	
ACE inhibitors	0	0	5	18	
Spironolactone	0	0	1	13	
Echocardiography^*e*^					
nLVIDd (cm)	1.43 ± 0.03	1.50 ± 0.04	1.84 ± 0.05	2.11 ± 0.08	<0.001
nLVIDs (cm)	0.87 ± 0.04	0.86 ± 0.03	0.90 ± 0.04	1.00 ± 0.06	0.12
nLAD (cm)	0.98 ± 0.03	1.02 ± 0.03	1.32 ± 0.05	1.52 ± 0.07	<0.001
LA/Ao	1.38 ± 0.04	1.40 ± 0.05	1.77 ± 0.08	2.27 ± 0.1	<0.001

a ACVIM, American College of Veterinary Internal Medicine. Continuous variables are reported as means ± standard errors.

b *P* values are from ANOVA tests.

c BCS, body condition score.

d Eleven group A dogs, 9 B1 dogs, and 1 C/D dog had missing information on cardiac medication. ACE, angiotensin-converting enzyme.

e Three group A dogs did not have echocardiographic data. nLVIDd, normalized left ventricular internal diameter end diastole; nLVIDs, normalized left ventricular internal diameter end systole; nLAD, normalized left atrial diameter; LA/Ao, left atrial to aortic root diameter ratio.

A total of 528 operational taxonomic units (OTUs) with known taxonomic lineages were identified, and their abundances were calculated (see Table S1, tab 1, in the supplemental material). The abundances of taxa in the ranks of phylum, class, order, and family were also calculated based on the OTU abundances (Table S1, tab 2).

10.1128/mSystems.00111-21.1 TABLE S1 Tab 1, OTU table. Tab 2, Taxa table. Tab 3, Alpha diversity. Tab 4, Beta diversity. Tab 5, Bootstrap analysis of alpha diversity. Tab 6, Statistical analysis of taxa. Tab 7, Statistical analysis of OTUs. Tab 8, Dysbiosis index and fecal bacterial DNA (log_10_). Tab 9, Statistical analysis of bile acids. Tab 10, Statistical analysis of SCFAs. Tab 11, Correlations between DI and fecal bacteria. Tab 12, Correlations between DI, fecal bacteria, and BAs. Tab 13, Physical characteristics of 50 dogs with matching fecal and serum samples. Tab 14, MaAsLin2 analysis between significant circulating metabolites and OTUs. Tab 15, Correlations between OTUs and echo variables. Tab 16, Statistical analysis of fecal bile acids. Download Table S1, XLSX file, 0.4 MB. Copyright © 2021 Li et al. 2021 Li et al. https://creativecommons.org/licenses/by/4.0/ This content is distributed under the terms of the Creative Commons Attribution 4.0 International license.

### Alpha and beta diversities.

**FIG 1:**
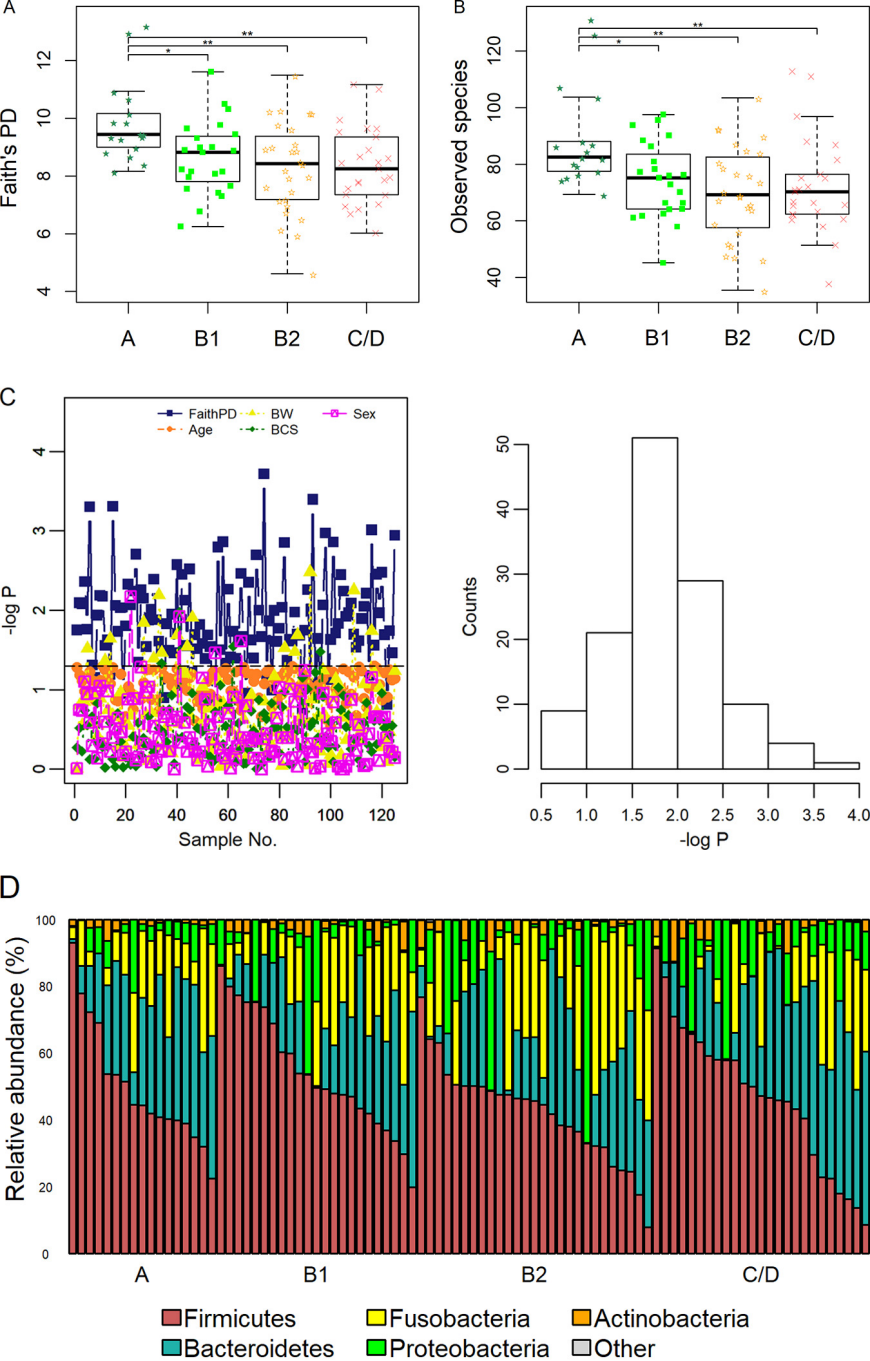
Faith’s phylogenetic diversity (PD) index (A) and number of unique bacterial species (B). *P* values were from Tukey’s *post hoc* tests following ANOVA. (C) Bootstrap experiments. Only subsamples with no difference in age are shown. Distributions of *P* values on Faith’s PD index, body weight (BW), age, body condition score (BCS), and sex from a bootstrap study (left) and histogram of *P* values on Faith’s PD index (right). *P* values are expressed as −log_10_ *P*. (D) Bar plots of the five predominant phyla. *, *P* < 0.05; **, *P* < 0.01.

The alpha diversity indexes based on the Faith’s phylogenetic diversity (PD) metric and the number of distinct species were calculated (Fig. 1A and B and Table S1, tab 3). Significant differences in both indexes were observed between group A and the three MMVD groups (adjusted *P* < 0.05 in all cases).

**FIG 2:**
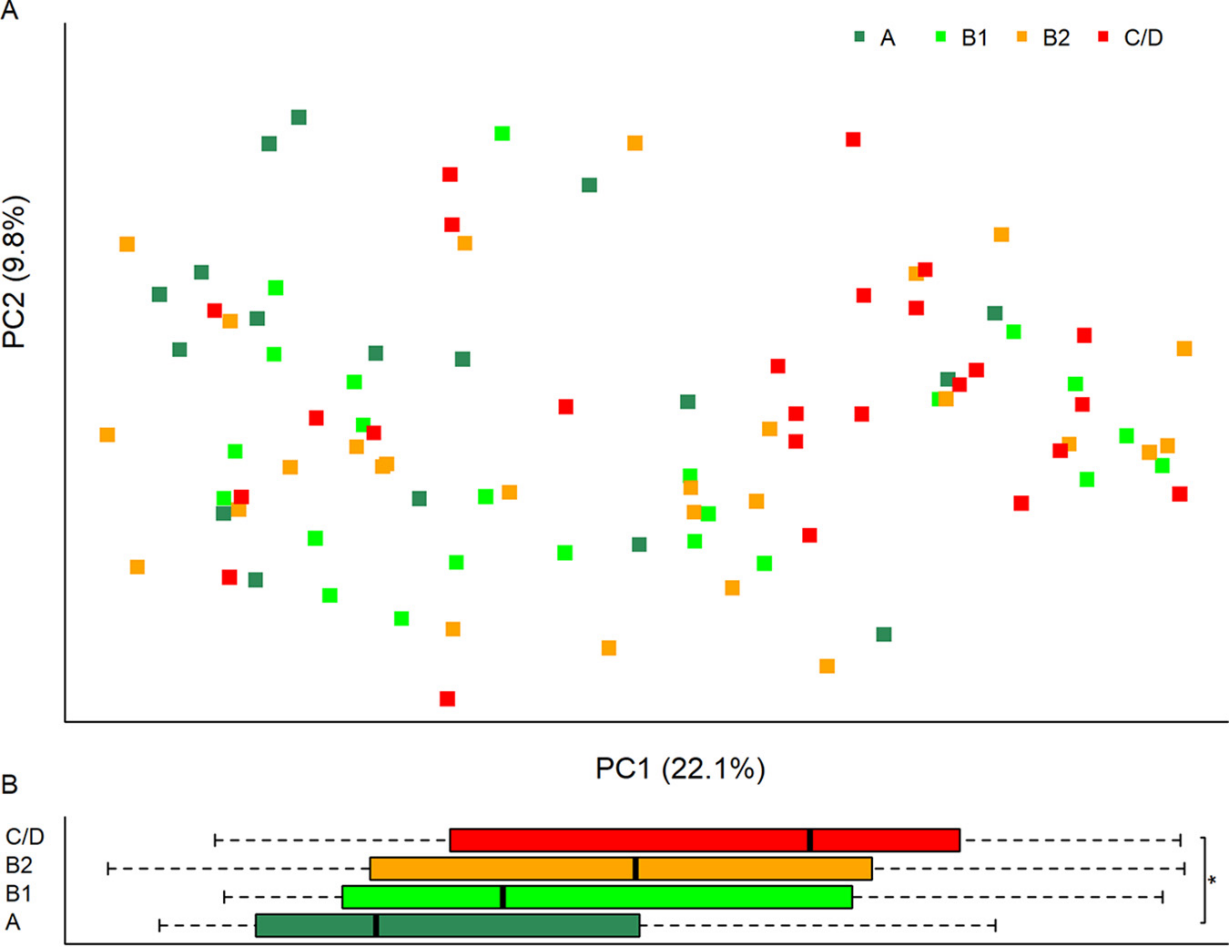
(A) Principal-coordinate analysis on the Bray-Curtis distance of the four groups. The first two principal coordinates (PCs), PC1 and PC2, are shown. The *x* and *y* axes indicate data variances captured by PC1 and PC2, respectively. (B) Box plots of PC1 by groups. ANOVA and Tukey’s tests found differences on PC1. No difference was found on PC2. Adjusted P value: *, *P* < 0.05.

Significant changes on the Bray-Curtis (BC) distances were observed among the groups using the permutational multivariate analysis of variance (PERMANOVA) test (*P* = 0.008) (Fig. 2A; Table S1, tab 4). Differences were observed between group A versus group B2 and versus group C/D (PERMANOVA, *P* = 0.013 and 0.005, respectively). But the difference between group A and group B1 did not reach statistical significance (*P* = 0.06). On principal coordinate 1 (PC1), which accounted for 22.1% of the data variance, a difference between group A and group C/D was observed (adjusted *P* = 0.038) (Fig. 2B). No difference was found on PC2.

No difference in alpha diversity or beta diversity was observed between groups B1, B2, and C/D.

### Age effect on alpha diversity.

To test the hypothesis that the observed diversity differences were independent of age difference, bootstrap subsampling was performed. In one simulation, 125 bootstrapped subsamples with no age difference were identified (*P*_age_ > 0.05). Of those, significant changes in Faith’s PD were observed in 85.6% (107/125) of the bootstrapped data sets (*P* < 0.05 in all cases) (Table S1, tab 5), with the median *P* value of 0.018 (IQR, 0.007 to 0.031) (Fig. 1C).

### Taxonomical differences.

First, we examined the overall changes in taxa. No significant change in abundance was observed in any taxonomical rank (Table S1, tab 6, and Fig. S1). The five predominant phyla, *Firmicutes*, *Bacteroidetes*, *Fusobacteria*, *Proteobacteria*, and *Actinobacteria*, accounted for more than 99% of the total bacteria (Fig. 1D; Fig. S1A). *Fusobacteria* was increased in groups B1 and B2 but decreased in group C/D compared to that in group A. However, these changes did not reach statistical significance. At the family level, *Paraprevotellaceae* were decreased while *Actinomycetaceae* were increased (false-discovery rate [FDR] = 0.07 in both cases) (Fig. S1B).

10.1128/mSystems.00111-21.3 FIG S1 Relative abundances of phyla (A) and families (B). Download FIG S1, PDF file, 0.1 MB. Copyright © 2021 Li et al. 2021 Li et al. https://creativecommons.org/licenses/by/4.0/ This content is distributed under the terms of the Creative Commons Attribution 4.0 International license.

**FIG 3:**
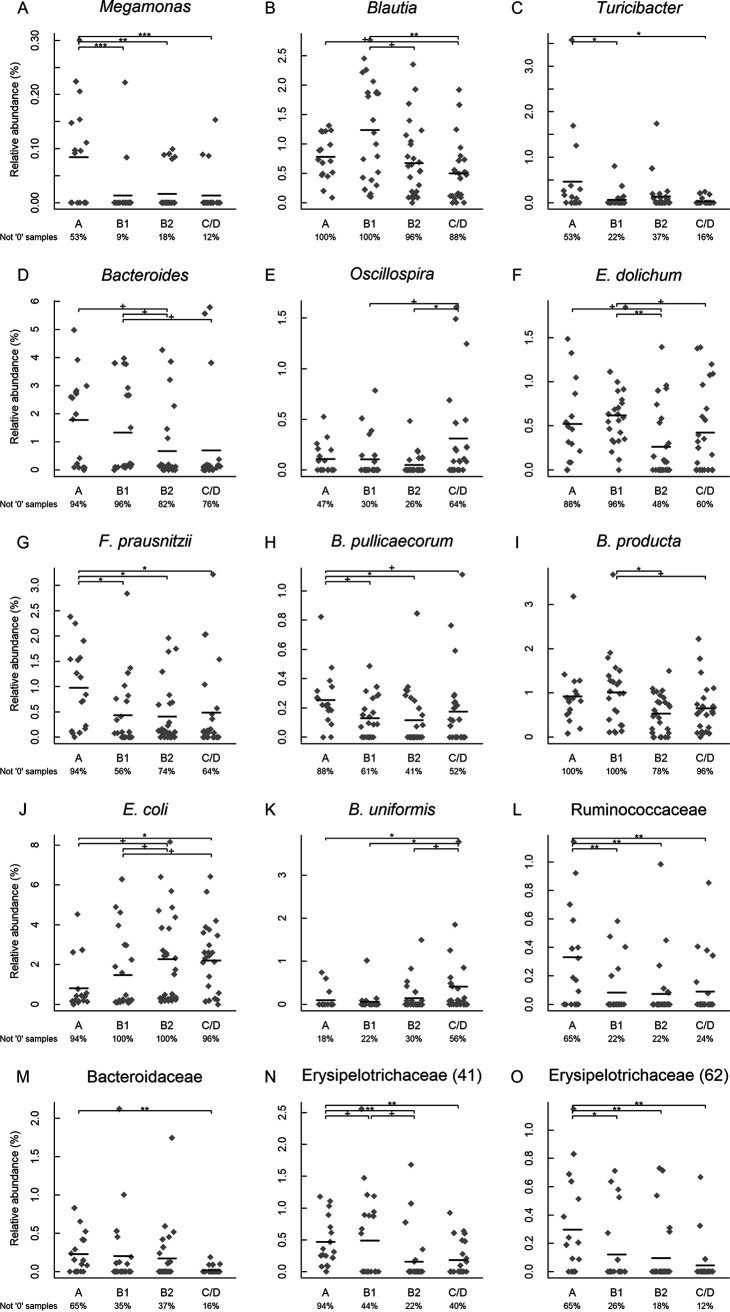
Significant operational taxonomical units (OTUs). Bacteria that shared at least 97% sequence similarity in their 16S marker genes were considered to be the same OTU. The lowest taxonomical rank in the OTU lineage is shown, with its OTU identifier (ID) inside the parentheses. These OTUs represented five genera, *Megamonas*, *Blautia*, *Turicibacter*, *Bacteroides*, and *Oscillospira* (A to E), six species, *E. dolichum*, *F. prausnitzii*, *B. pullicaecorum*, *B. producta*, E. coli, and *E. uniformis* (F to K), and three families, *Ruminococcaceae*, *Bacteroidaceae*, and *Erysipelotrichaceae* (L to O). N and O were two different OTUs. The horizontal lines indicated means. The percentage of nonzero samples is indicated below each group. Nonparametric Kruskal-Wallis test was performed on each OTU. Significant OTUs were subject to *post hoc* Dunn’s tests with corrections for false-discovery rate (FDR). **^+^**, FDR < 0.1; *, FDR < 0.05; **, FDR < 0.01; ***, FDR < 0.001.

Second, we examined individual OTUs. Fifteen significant OTUs were identified (*P* < 0.05, *q* < 0.20 in all cases) (Table S1, tab 7). Among them are six species, five genera, and three families. The abundances of *Megamonas*, *Blautia*, *Bacteroides*, and *Turicibacter* were decreased in MMVD dogs versus those in healthy dogs, while *Oscillospira* was increased (Fig. 3A to E). At the species level, Eubacterium dolichum, Faecalibacterium prausnitzii, Blautia producta, and Butyricicoccus pullicaecorum had reduced abundances, while Escherichia coli and Bacteroides uniformis had increases in MMVD dogs compared to that in healthy dogs (Fig. 3F to K). The abundance of *Ruminococcaceae* was increased while those of *Bacteroidaceae* and *Erysipelotrichaceae* were decreased (Fig. 3L to O).

### Fecal DI, BA, and SCFA.

**FIG 4:**
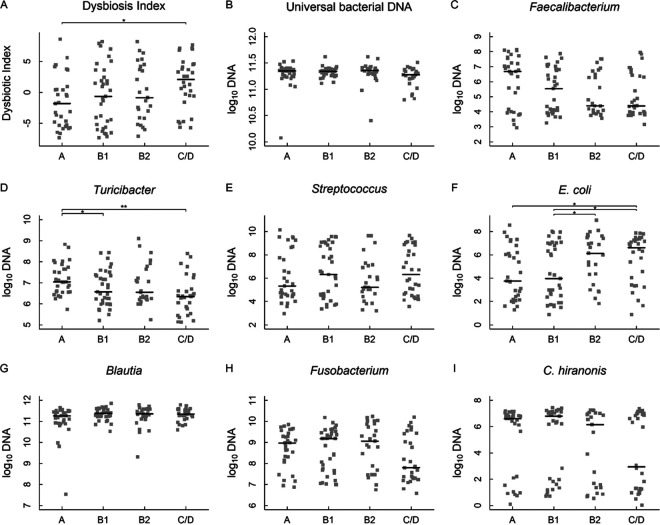
PCR-based fecal dysbiosis index using a panel of gut bacteria. Dysbiosis index (A), total bacteria (B) and seven bacterial genera and species, *Faecalibacterium*, *Turicibacter*, *Streptococcus*, E. coli, *Blautia*, *Fusobacterium*, and *C. hiranonis* (C to I) in dogs with MMVD. Bacterial abundances were measured using quantitative PCR. The horizontal lines denoted medians. Adjusted *P* values: *, *P* < 0.05, **, *P* < 0.01.

The fecal samples from 121 dogs, including 85 (85/92) from the 16S sequencing study, were included for the quantitative PCR (qPCR)-based dysbiosis (DI) analysis (Fig. 4; Table S1, tab 8). The average DIs for groups A, B1, B2, and C/D were −1.48, −0.6, 0.01, and 1.47, respectively (Kruskal-Wallis test, *P*_K-W_ = 0.07) (Fig. 4A). The difference between group A and group C/D was significant (adjusted *P* = 0.034). Similar to the 16S rRNA sequencing data, the abundances of *Turicibacter* and E. coli were different (P_K-W_ = 0.007 and 0.025, respectively). *Turicibacter* was more abundant in group A than in groups B1 or C/D (adjusted *P* = 0.035 and 0.002, respectively) (Fig. 4D), while E. coli abundance was greater in group C/D than in group A or group B1 and greater in group B2 than in group B1 (adjusted *P* = 0.032, 0.026, and 0.043, respectively) (Fig. 4F). The abundances of total bacteria appeared to be equal between the groups (Fig. 4B).

No difference was observed in fecal BAs except glyco-CA (*P*_K-W_ = 0.008) (Table S1, tab 9). Group B2 had a greater amount of glycol-CA than group A or group B1 (adjusted *P* = 0.002 and 0.024, respectively). No difference was found in fecal SCFAs (Table S1, tab 10).

**FIG 5:**
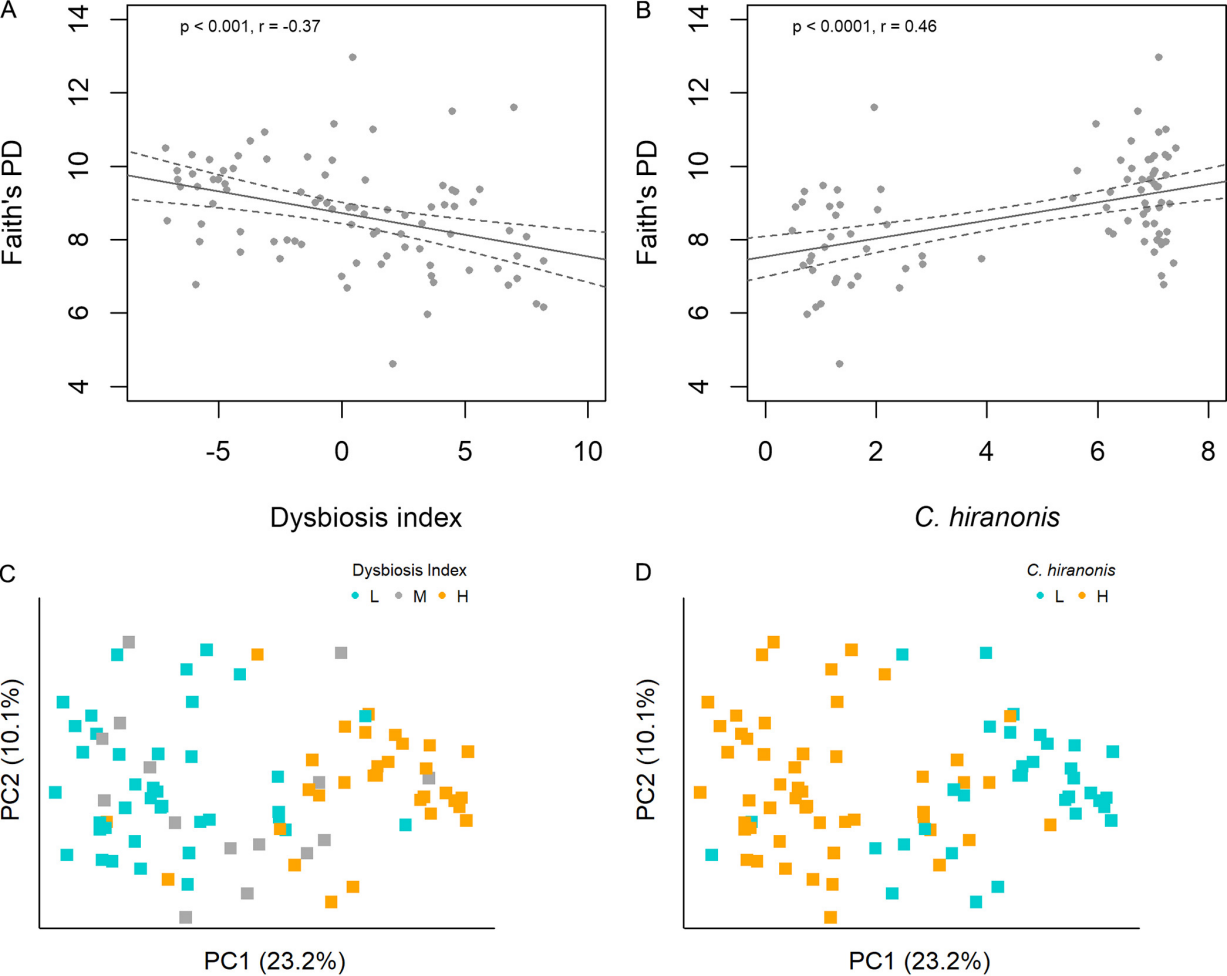
Alpha and beta diversity analyses on DI and *C. hiranonis* using the 16S sequencing data. Pearson’s correlations between Faith’s PD index and DI (A) and *C. hiranonis* (B). Principal-coordinate analysis using the Bray-Curtis distances on DI (C) and *C. hiranonis* (D). Samples were colored based on DI, cyan (L, DI ≤ 0), gray (M, 0 < DI < 2), or orange (H, DI ≥ 2) (C), and on *C. hiranonis* abundance, cyan (L, log_10_ DNA < 4.5) or orange (H, log_10_ DNA ≥ 4.5) (D). The first two principal coordinates, PC1 and PC2, are displayed with the percentages of data variation denoted in the *x* and *y* axes, respectively. (A and B) Fitted linear regression lines with 95% confidence intervals are included. Clustering of samples along PC1 was evidenced in panels C (*P* = 1.9e^−14^) and D *P* = 8.5e^−14^. DI, dysbiosis index.

Faith’s PD index showed a negative correlation with DI but a positive one with Clostridium hiranonis (*P* = 0.0006 and 6e^−6^; *r* = −0.37 and 0.47, respectively) (Fig. 5A and B). The BC distance-based plots showed visible clustering of samples along PC1 by DI or *C. hiranonis* abundance (*P* = 1.9e^−14^ and 8.5e^−14^, respectively) (Fig. 5C and D). No difference was observed on PC2 (*P* > 0.7).

### Correlations between DI, BAs, and bacterial abundances.

DI was positively associated with *Streptococcus* and E. coli but negatively associated with *Faecalibacterium*, *Fusobacterium*, and *C. hiranonis* (|*r*| > 0.6, *P* < 1e^−6^ in all cases) (Fig. S2A to E and Table S1, tab 11).

10.1128/mSystems.00111-21.4 FIG S2 Correlations between dysbiosis index and fecal bacteria. Download FIG S2, TIFF file, 8.8 MB. Copyright © 2021 Li et al. 2021 Li et al. https://creativecommons.org/licenses/by/4.0/ This content is distributed under the terms of the Creative Commons Attribution 4.0 International license.

**FIG 6:**
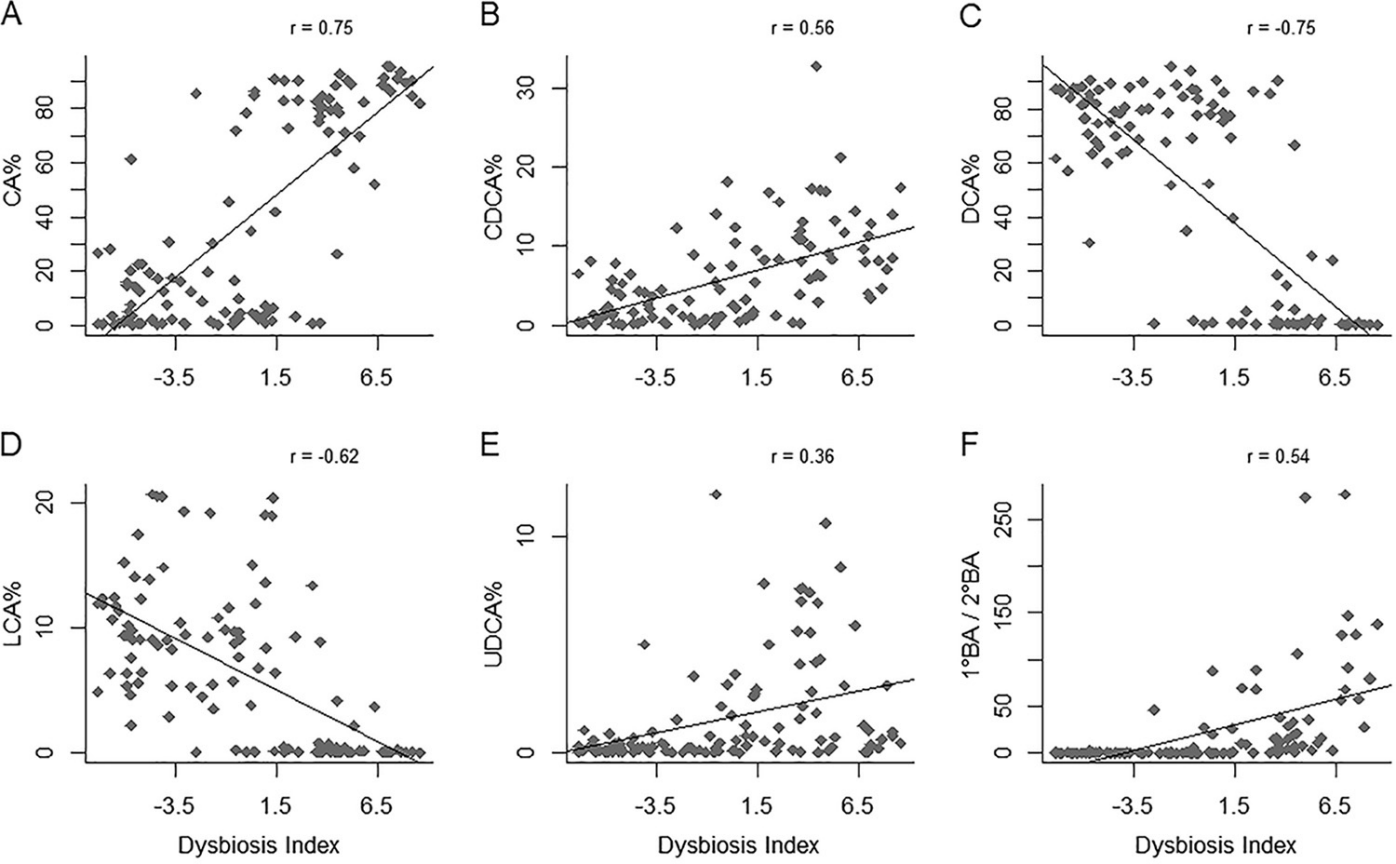
Pearson’s correlations between fecal bile acids (BAs) and dysbiosis index. CA, cholic acid; CDCA, chenodeoxycholic acid; DCA, deoxycholic acid; LCA, lithocholic acid; UDCA, ursodeoxycholic acid; 1° BA/2° BA, the ratio of primary to secondary BAs. The percentage of each BA was calculated as the ratio of the BA to the sum of primary and secondary BAs. Only unconjugated BAs were considered. Correlation coefficients (*r*) were indicated on the top right corner. *P* < 1e^−4^ in all cases.

The fecal DI was positively associated with two primary BAs, CA and CDCA, (*P* < 1.5e^−10^ in both cases; *r* = 0.75 and 0.56, respectively) (Fig. 6A and B, and Table S1, tab 12). The DI was negatively correlated with two secondary BAs, DCA and LCA (*P* < 3.4e^−13^ in both cases; *r* = −0.62 and −0.75, respectively) (Fig. 6C and D), but positively correlated with UDCA (*P* = 7.8e^−5^, *r* = 0.36) (Fig. 6E). The ratio of primary BA to secondary BA (1°/2°) had a positive association with DI (*P* = 8.8e^−10^; *r* = 0.54) (Fig. 6F).

**FIG 7:**
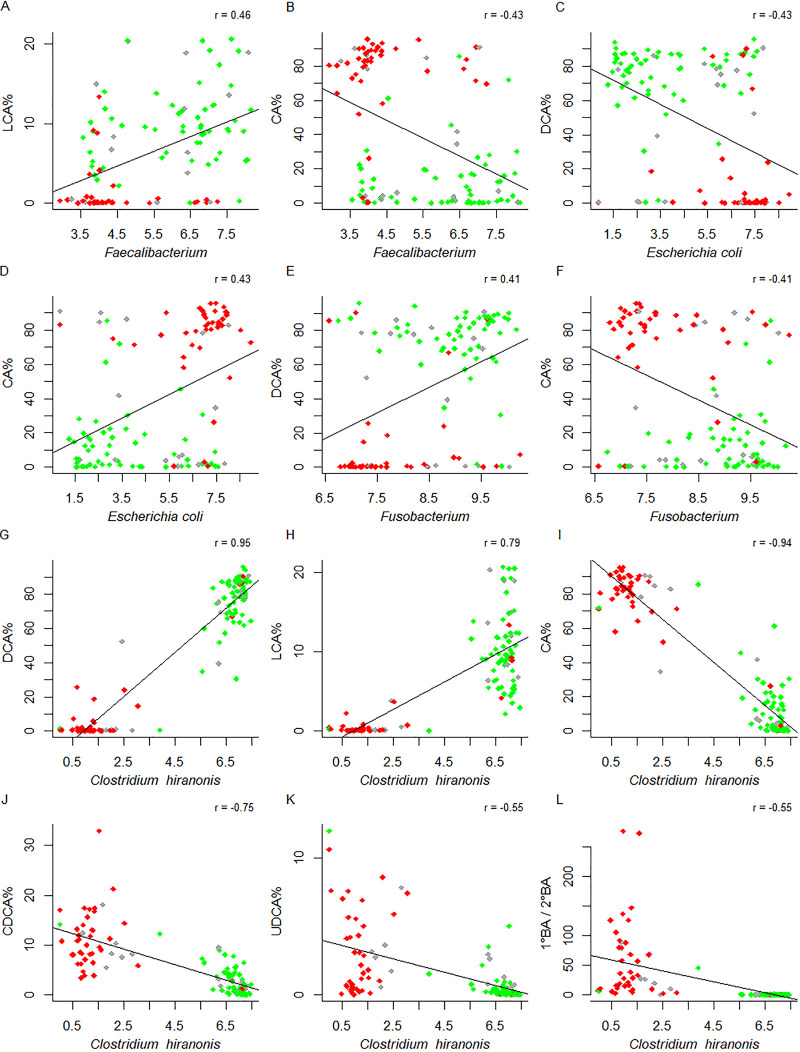
Pearson’s correlations between fecal bile acids (BAs) and gut microbes. CA, cholic acid; LCA, lithocholic acid; DCA, deoxycholic acid; CDCA, chenodeoxycholic acid; UDCA, ursodeoxycholic acid; 1° BA/2° BA, the ratio of primary to secondary BAs; *r*, correlation coefficient. (A to L) The percentage of each BA was calculated as the ratio of BA to the sum of primary and secondary BAs (*y* axis). Bacterial abundance was expressed as log_10_ DNA abundance (*x* axis). Only unconjugated BAs were considered. Samples were colored by dysbiosis index (DI): green for DI ≤ 0, red for DI ≥ 2, gray for others. *P* < 1e^−5^ in all cases.

*Faecalibacterium*, E. coli, and *Fusobacterium* showed modest to moderate associations with BAs (*P* < 0.0001 in all cases) (Fig. 7A to F, green for DI ≤ 0, red for DI ≥ 2, gray for 0 < DI < 2; Table S1, tab 12). While E. coli was positively associated with primary BA but negatively associated with secondary BA, the opposite was observed for *Faecalibacterium* and *Fusobacterium*. Strong associations between *C. hiranonis* and BAs were observed. While positive associations with the secondary bile acids DCA and LCA were observed (*P* = 2.2e^−16^ in both cases) (Fig. 7G and H), *C. hiranonis* was inversely correlated with the primary bile acids CA and CDCA, the secondary bile acid UDCA, and 1°/2° (*P* < 5.4e^−10^ in all cases) (Fig. 7I to L). Notably, when the log_10_ abundance of *C. hiranonis* was below the threshold of 4.5, little conversion from primary BAs to secondary BAs took place (Fig. 7G to L).

### Correlations between OTUs, serum metabolites, and echo variables.

Fifty dogs had matching serum samples in the previously published metabolomics study (Table S1, tab 13) (7). Generalized linear models identified several significant but weak correlations between the OTUs and metabolites (|*r*| ≥ 0.2, FDR ≤ 0.05) (Table 2; Table S1, tab 14). Remarkably, *Megamonas* showed positive associations with 7 short-chain acylcarnitines and carnitine, while *Lactobacillus* was positively correlated with 6 short-chain acylcarnitines. A negative correlation was observed between *Erysipelotrichaceae* and TMAO.

**TABLE 2:**
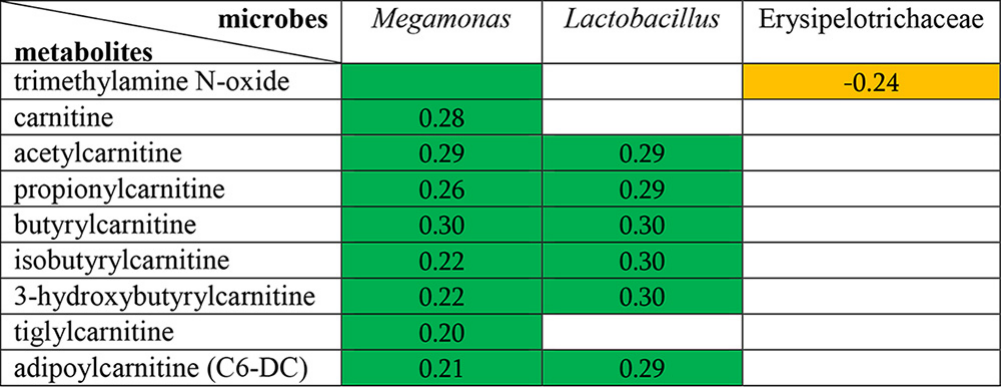
Correlations between gut microbes and circulating carnitine and short-chain acylcarnitines (C_2_ to C_6_)^*a*^

a Green color indicates a positive correlation, while orange color indicates a negative correlation between the corresponding microbes and metabolites. All correlations had adjusted *P* values of ≤0.05 and absolute values of correlation coefficient of ≥0.2. The analysis was performed using the multivariate statistical framework implemented in the R package MaAsLin2.

No correlation was found between OTUs and the three key echo variables after adjusting for multiple testing errors (FDR > 0.05 in all cases) (Table S1, tab 15).

### Serum BAs.

No BA met the stringent selection criteria in the serum metabolomics profiling study (7). The mean concentrations (in relative quantification) of CDCA were 1.04, 0.77, 0.95, and 1.39 for groups A, B1, B2, and C/D, respectively (*P* = 0.07) (Table S1, tab 16), and was higher in group C/D than in group B1 (adjusted *P* = 0.046).

## DISCUSSION

A limited number of studies have examined the roles of gut microbiota in heart failure (HF) in humans and animal models (20–25). Emerging evidence causally links gut microbiota to atherosclerotic disease, but observations between gut bacteria and HF remain associative (26). To our knowledge, no study has documented gut microbiota changes in the preclinical phases leading to HF. In this study, we compared gut microbiome profiles in MMVD dogs at preclinical B1 and B2 stages and those with histories of CHF with that of healthy dogs. Alpha diversity, which describes the number (richness) or distribution (evenness) of different bacterial species in the gut, was greater in the healthy dogs than in dogs with MMVD, but no difference was found among the three MMVD groups. So far, two studies reported reduced alpha diversities in human HF patients and in a rodent model with induced HF (24, 25), but other studies showed no change in alpha diversity between HF subjects and healthy controls in humans or dogs (20–23). We also observed nominal differences in beta diversity, which measures similarity of different microbial groups. Although it is difficult to fully understand the changes and assess the discrepancies with only a small number of microbiome studies on HF, our results clearly showed that shifts in gut microbiota began at the very early preclinical stages when dogs had little or no evidence of cardiac remodeling. Initially, we expected to see differences in gut microbial diversity among the three MMVD groups. Although both stage B1 and B2 dogs are asymptomatic of CHF, stage B2 dogs exhibit evidence of more advanced disease with hemodynamically significant mitral valve regurgitation, as evidenced by radiographic and echocardiographic findings of cardiomegaly. The stage C dogs are characterized as having exhibited past or current clinical signs of CHF secondary to MMVDs. The current “leaky gut” hypothesis postulated bowel wall edema and impaired intestinal barrier function due to heart failure (9). To date, no report on gut microbiota in preclinical patients before the onset of HF is available. It was recently reported that levels of circulating uremic toxins, including TMAO and other nitrogenous wastes, were increased in dogs with preclinical MMVD versus that in healthy dogs (7). We thus expand the current hypothesis that the changes in gut microbiota in the preclinical stage may have already compromised the integrity of intestinal barrier function to a degree that the resultant “leaked” gut-derived metabolites trigger an initial inflammatory response, leading to progressive worsening of MMVD.

There is a trade-off between sensitivity and specificity in high-dimensional data analysis. While it is important to adjust for multiple testing, a stringent *P* value threshold may also increase false-negative calls. In a recent untargeted metabolomics study that linked gut flora metabolism and cardiovascular disease, l-carnitine was not on the top tier of metabolites that met the stringent *P* value cutoff but was later identified using less stringent criteria (8, 12). As a hypothesis-driven study, we decided to relax the adjusted *P* value (*q* value) threshold to 0.20. We identified changes in five genera and six species, including E. coli and *Turicibacter* (*P* < 0.05, *q* = 0.16 in both cases), both of which were confirmed by qPCR (*P* = 0.025 and 0.007, respectively). *Turicibacter*, a genus in the phylum *Firmicutes*, is commonly found in the gastrointestinal (GI) tracts of animals. This bacterium influences the host’s physiological processes by modulating certain gut microbe-dependent hormones (27–29). Specifically, Turicibacter sanguinis, one of the spore-forming gut microbes that were shown to signal gut cells to increase serotonin (5-HT) production, expresses a protein homologous to mammalian 5-HT transporter and is able to import 5-HT into the cell (28, 29). Genetic interactions between *Turicibacter* sp. and bile acids were also reported (27). The 5-HT signaling pathway has been implicated in the development and progression of canine MMVD (30). Previous studies reveal an association between serum 5-HT concentration and disease state in which dogs at high risk for MMVD as well as dogs in early stages of MMVD exhibit increased serum 5-HT, whereas dogs with end-stage MMVD exhibit decreased serum 5-HT (31, 32). The potential association between *Turicibacter*, 5-HT signaling, and MMVD pathogenesis warrants further investigation. *Bacteroides*, *Blautia*, and *Megamonas* all express enzymes for propionate production pathways and are normally found in the guts of healthy carnivores (33–37). *Oscillospira*, a genus of commensal bacteria that produces butyrate and is commonly found in healthy guts, appeared to confer protection against atherosclerosis and reduce plaque size in a mouse study (38–41). *F. prausnitzii* and *B. pullicaecorum*, both members of the *Ruminococcaceae* family and clostridial cluster IV, produce a high level of butyrate with promising probiotic potentials (35, 36). *E. dolichum* of the *Erysipelotrichaceae* family also produces butyrate and acetate (40). Butyrate and propionate possess well-known anti-inflammatory effects (42). The reduced abundances of these bacterial genera and species in dogs with MMVD suggested a net decrease in SCFA production in the gut and a reduced protection against inflammation. In addition, SCFAs are important signaling molecules which regulate diverse biological processes, including cardiovascular disease (9, 11, 19). However, no difference was observed in fecal SCFAs in our study. It was reported that 90% to 95% SCFAs produced in the colon were reabsorbed by the gut mucosa (43). Thus, the concentrations of fecal SCFAs may not accurately reflect those produced by gut microbiota. More studies are needed to explore whether the changes in these SCFA-producing bacteria have any effect on the levels of SCFAs in circulation.

A search for TMA-producing genes in microbial metagenomes showed that the E. coli genome displayed ∼99% identities to the gene for carnitine oxygenase (*cntA*), the key gene in the main TMA synthesis pathway (44). Prior studies also reported increased E. coli abundances in human and canine HF patients compared to that in healthy controls (20, 24). The observations that circulating TMAO concentrations were increased in dogs with B2 stage MMVD and CHF (6, 7), and that E. coli abundance was higher in B2 and CHF dogs than in B1 or healthy dogs, underscored a potential involvement of E. coli in TMAO production.

The dysbiosis index is a PCR-based tool that allows quantifications of gut dysbiosis using a panel of eight selected bacterial groups (45). Recent studies showed that DI was increased in dogs with inflammatory bowel disease (IBD) (46–48). We tested the hypothesis that DI increased with the severity of MMVD. Indeed, our data showed that the average DI progressively increased from −1.48 in group A, to −0.6, 0.01, and 1.47 in groups B1, B2, and C/D, respectively, and that the difference between groups A and C/D reached statistical significance (adjusted *P* < 0.05). Notably, there were considerable within-group variations. It is possible that a larger sample size may further improve statistical power. Nevertheless, this PCR-based DI tool showed promises to monitor MMVD onset and progression and offered an opportunity to improve its sensitivity and specificity with the selection of a cardiac-specific bacterial panel. The qPCR results validated the significant differences observed for *Turicibacter* and E. coli, and similar trend for *F. prausnitzii* but not for *Blautia*, from the 16S rRNA gene sequencing. Four bacteria showed moderate to strong correlations with DI: *Streptococcus* and E. coli had positive associations, while *Fusobacterium* and *C. hiranonis* had negative ones (*P* < 1e^−10^, |*r*| ≥ 0.6 in all cases). Our results demonstrated a progressive increase in gut microbiota dysbiosis in MMVD dogs.

Gut microbiota influences host metabolism and physiology by producing numerous metabolites. Conversely, these metabolites reshape the structure and composition of gut microbiota (49). We further investigated the relationships among BAs, DI, and gut microbes. Bile acids have received considerable attention recently due to their ability to regulate many physiological processes as signaling molecules. Primary BAs are converted to secondary BAs by gut bacteria whose genomes contain the BA-inducible operons (*bai*) with 7α/β-dehydroxylase activities (50). Secondary BAs are thought to render protections against the growth of several pathogens, including Clostridium difficile, E. coli, and Clostridium perfringens (51–53). Deoxycholic acid, a secondary BA, reduces accumulations of inflammatory mediators in ileum, and supplementation of DCA in diet diminished C. perfringens-induced severe inflammatory effect and body weight loss in chickens (54). Interestingly, one recent study reported increased ratio of plasma secondary/primary BA ratio in 142 chronic HF patients compared with that in 20 healthy control subjects (55), but it is difficult to draw any meaningful conclusion with the sole observation. *C. hiranonis*, one of the *Clostridium* spp. that possess 7α-hydroxylation ability (56), was one of the bacteria in the DI panel. Remarkably, when *C. hiranonis* abundance (in log_10_) was >4.5, essentially all primary BAs were converted to secondary BAs (1° BA/2° BA ≈ 0) (Fig. 7L), suggesting that, when abundant, *C. hiranonis* is sufficient to convert the majority, if not all, of primary BAs to secondary BAs. Furthermore, our data suggested that secondary BAs inhibited the growth of E. coli but promoted that of *Fusobacterium* and *Faecalibacterium* (Fig. 7A to F).

Positive correlations were found between circulating short-chain acylcarnitines and gut bacteria, *Lactobacillus* and *Megamonas*. The abundance of *Megamonas* was reduced in MMVD dogs compared to that in healthy dogs. Acylcarnitines, key intermediates of long-chain-FA transport and oxidation, accumulate in circulation as a result of incomplete or inefficient fatty acid oxidation and have been used as diagnostic markers for disorders in peroxisomal or mitochondrial oxidation processes (57–59). Elevated long-chain acylcarnitines in circulation were documented in human HF patients compared to that in normal ones (60, 61). Accumulation of long-chain acylcarnitines was thought to contribute to HF by stimulating reactive oxygen species (ROS) production and releasing circulating inflammatory mediators (61). In dogs, the severity of MMVD was correlated with the concentrations of both short-chain and long-chain acylcarnitines (7), some of which were reduced in response to diet intervention with demonstrated clinical benefits (62, 63). In addition, a negative association with TMAO was observed with the *Erysipelotrichaceae* family, whose abundance was reduced in MMVD dogs. Despite the significance (FDR < 0.05 in all cases), the associations between these gut bacteria and circulating metabolites were weak. It is important to note that only 50 samples had both fecal rRNA gene sequencing and serum metabolomics data and that there were only 6 dogs (6/50) in the healthy control group. Nevertheless, this pilot experiment may offer an opportunity for future studies.

We explored, for the first time, the relationships between gut microbiome and gut-derived metabolites in dogs with all stages of MMVD. Because these dogs were privately owned, we were unable to rule out potential cofounding effects from diet and breed. In addition, all C/D dogs and the majority of the B2 dogs were on one or more common cardiac medications at the time of the study. Although the bootstrapping study supported the hypothesis that the changes were not due to age difference, we were unable to completely rule out the possibility of a small confounding effect from age. Significantly, our study expands the current “gut hypothesis” to include gut dysbiosis at the preclinical stages when there is little or no evidence of cardiac remodeling and lays a foundation for future microbiome research in canine and human MMVD.

## MATERIALS AND METHODS

### Animals and study approval.

The study protocol was reviewed and approved by the University of Pennsylvania Institutional Animal Care and Use Committee, and informed owner consent was obtained. Clinically healthy dogs 7 years of age or older without a heart murmur and without concurrent systemic disease were prospectively enrolled as the control group (group A). This group of dogs primarily consisted of systemically healthy dogs owned by students and staff of the hospital. A cohort of dogs 7 years of age or older with a left apical systolic murmur and echocardiographic (echo) diagnosis of thickened and prolapsing mitral valve leaflet(s) and mitral regurgitation as well as clinical history and physical exam consistent with stage B1, B2, C, or D MMVD were considered for groups B1, B2, and C/D, respectively (3). Any dog with severe concurrent systemic disease, including diabetes mellitus, cancer, or renal failure, or those with any congenital heart disease were excluded. Dogs with signs of gastrointestinal illness such as vomiting or diarrhea and those that had received antibiotics within 30 days were also excluded.

### Echocardiography.

Echo studies (iE33; Philips Healthcare, Andover, MA) were performed without sedation. Left ventricular internal dimensions in end-diastole (nLVIDd) and end-systole (nLVIDs), left atrial diameter (nLAD), and aortic root diameter (nAoD) were measured from right parasternal short axis 2-dimensional images and normalized to body weight (64). The ratio of the left atrial diameter to the aortic root diameter (LA/Ao) was calculated.

### Fecal 16S rRNA gene sequencing.

Dog owners were instructed to collect a fresh fecal sample on the day of their pet’s appointment. The sample was divided in aliquots and frozen at −80°C until analysis was performed.

Fecal genomic DNA extractions were performed using PowerFecal DNA isolation kit (Mo Bio Laboratories) and quantified by Quant-It Pico Green (Thermo Fisher Scientific) according to manufacturers’ protocols. The 16S rRNA gene library was constructed according to Illumina’s 16S metagenomic sequencing library preparation guide. Sequencing on the V3-V4 region of the 16S gene was performed in an Illumina MiSeq machine with 2 × 250 cycles as previously described (65). The sequences for the 16S amplicon PCR forward and reverse primers were 5′-TCGTCGGCAGCGTCAGATGTGTATAAGAGACAGCCTACGGGNGGCWGCAG and 5′-GTCTCGTGGGCTCGGAGATGTGTATAAGAGACAGGACTACHVGGGTATCTAATCC, respectively.

The bioinformatics pipeline for sequence analysis, alpha diversity, and beta diversity were described previously (65) and can also found in Text S1 in the supplemental material. Alpha diversity indexes of observed species, and Faith’s phylogenetic diversity (PD) and beta diversity indexes based on the Bray-Curtis (BC) metric were calculated at the rarefaction depth of 7,000 sequences. Sequences that shared a minimum of 97% of identity were clustered into an operational taxonomic unit (OTU), and a taxonomical lineage was assigned to each OTU by searching the Greengenes database (August 2013 release).

10.1128/mSystems.00111-21.5 TEXT S1 Supplemental methods for fecal bile acid analysis. Download Text S1, DOCX file, 0.03 MB. Copyright © 2021 Li et al. 2021 Li et al. https://creativecommons.org/licenses/by/4.0/ This content is distributed under the terms of the Creative Commons Attribution 4.0 International license.

### DI, BA, and SCFA.

Fecal samples from additional dogs were collected and included in these analyses. Fecal genomic DNA (100 mg) was shipped to Texas A&M University GI Lab (College Station, TX) for the quantitative PCR analysis to measure the DNA abundances of eight bacterial groups: total bacteria, *Faecalibacterium*, *Turicibacter*, *Streptococcus*, Escherichia coli, *Blautia*, *Fusobacterium*, and Clostridium hiranonis. The PCR primers and protocol and method for dysbiosis index (DI) calculation were described in detail previously (45). Bacterial DNA abundances were transformed using the logarithm with base 10.

Each fecal sample was split into two aliquots, one of which was shipped to Metabolon, Inc. (Morrisville, NC) for the targeted BA analysis, including 2 primary BAs (CA and CDCA) and 3 secondary BAs (DCA, LCA, and UDCA). The panel also included 10 glycine- and taurine-conjugated primary and secondary BAs. The other set of aliquots was shipped Texas A&M GI Lab (College Station, TX) for 4 straight-chain and 2 branched-chain short-chain fatty acid (SCFA) assays. The quantitation of BAs was performed using an Agilent 1290 Infinity/Sciex QTRAP 6500 liquid chromatography-tandem mass spectrometry (LC-MS/MS) system equipped with a C_18_ reverse-phase ultrahigh-performance liquid chromatography (UHPLC) column (Text S1; Table S2). The concentrations of SCFAs were measured using a stable isotope dilution gas chromatography (GC)/MS assay using the Agilent 6890N/Agilent 5975C system with a C_18_ solid-phase extraction column (47, 66).

10.1128/mSystems.00111-21.2 TABLE S2 QC accuracy for bile acids. Download Table S2, DOCX file, 0.03 MB. Copyright © 2021 Li et al. 2021 Li et al. https://creativecommons.org/licenses/by/4.0/ This content is distributed under the terms of the Creative Commons Attribution 4.0 International license.

The total amount of primary BAs was calculated by summing the concentrations of CA and CDCA, while that of secondary BAs was the sum of DCA, LCA, and UDCA. The percentage of each BA was calculated by dividing the concentration of the BA by the sum of all unconjugated BAs. The ratio of primary BAs to secondary BAs (1° BA/2° BA) was also calculated.

Alpha and beta diversity analyses were performed from the 85 samples that also had 16S sequencing data. Pearson’s correlation analysis was performed between Faith’s phylogenetic diversity (PD) index, DI, and *C. hiranonis* abundance. In addition, samples were trichotomized based on DI: L (DI ≤ 0), M (0 < DI < 2), and H (DI ≥ 2), or dichotomized based on the abundance of *C. hiranonis*: L (log_10_ DNA < 4.5) or H (log_10_ DNA ≥ 4.5). Analysis of variance (ANOVA) was performed using the first two principal coordinates, PC1 or PC2, as the dependent variable on the categorical DI or *C. hiranonis*.

### Statistical analysis.

Total taxa abundances at the taxonomical ranks of phylum, class, order, and family were calculated using the OTU table. The relative abundance for each OTU or taxon was calculated using the total sum scaling method and was further transformed using square root. OTUs or taxa with 0% relative abundance in more than 50% of the samples in every group were removed. Two group comparisons were performed using the Mann-Whitney test. For multiple groups, the nonparametric Kruskal-Wallis (K-W) analysis of variance was performed, and significant OTUs or taxa were subject to *post hoc* Dunn’s tests. The *P* values from both K-W tests and Dunn’s tests were adjusted for multiple testing error using the Benjamini-Hochberg (BH) method. Taxa or OTUs with adjusted *P* values of less than 0.10 were considered significant. Data normality was evaluated using the Shapiro-Wilk test. To compare alpha diversity, ANOVA and Tukey’s *post hoc* tests were performed. To compare beta diversity distances, permutational multivariate analysis of variance (PERMANOVA) was performed on the distance matrices with 1,000,000 permutations using the R package vegan (67). Principal-coordinate analysis (PCoA), an eigen-analysis of a dissimilarity matrix, was performed using the BC distance matrix. The first two principal coordinates (PCs), PC1 and PC2, were examined for their abilities to separate the groups using ANOVA and Tukey’s *post hoc* tests in which each PC was the dependent variable and group was the predictor variable. To compare the differences in DI, SCFA, and BA, K-W analysis and Dunn’s multiple comparisons were used.

### Correlation analyses.

Pearson’s correlation analysis was performed between DI, PCR-based bacterial abundances, and BAs. Dogs with the matching serum samples were identified from the previously published metabolomics study, which reported 173 differential metabolites (7). The adonis function in the R package vegan was used to evaluate how many variations in the BC distance can be explained by each of the 173 metabolites. *P* values were determined by 1,000 permutations and adjusted for multiple testing using the BH method. The Spearman correlation was performed to assess the association between the alpha diversity indexes and the metabolites and between OTUs and echo variables. The association between individual OTUs and metabolites was performed using general linear models implemented in the R package MaAsLin2, a multivariate statistical framework that finds associations between microbial community abundances and clinical metadata (68). The MaAsLin2 function was used with the following settings: low abundance OTUs with a minimal relative abundance of 0.01% in less than 10% of the samples were filtered. The proportional value of each OTU was transformed by taking the logarithm to the base 10. Age, body weight, and MMVD group were included as random effects. The BH method was applied for multiple-testing corrections. Correlations with adjusted *P* values of less than or equal to 0.05 and correlation coefficients greater than 0.2 or less than −0.2 were considered significant.

### Analysis of age effect.

To assess the potential age contribution to gut microbial diversity, 1,000 iterations of bootstrap resampling experiments without replacement were performed, where 12 samples from each group were randomly selected. The Faith’s PD indexes from each bootstrapped data set with no age difference were subject to ANOVA. The distributions of the *P* values on age, body weight, body condition score (BCS), sex, and Faith’s PD were analyzed.

## References

[1] Buchanan J. 1999. Prevalence of cardiovascular disorders, p 457–470. *In* Sisson D, Fox PR, Sydney M (ed), Textbook of canine and feline cardiology, 2nd ed. Saunders, Philadelphia, PA.

[2] Borgarelli M, Buchanan JW. 2012. Historical review, epidemiology and natural history of degenerative mitral valve disease. J Vet Cardiol 14:93–101. doi:10.1016/j.jvc.2012.01.011. 22386588

[3] Keene BW, Atkins CE, Bonagura JD, Fox PR, Haggstrom J, Fuentes VL, Oyama MA, Rush JE, Stepien R, Uechi M. 2019. ACVIM consensus guidelines for the diagnosis and treatment of myxomatous mitral valve disease in dogs. J Vet Intern Med 33:1127–1140. doi:10.1111/jvim.15488. 30974015 PMC6524084

[4] Oyama MA, Elliott C, Loughran KA, Kossar AP, Castillero E, Levy RJ, Ferrari G. 2020. Comparative pathology of human and canine myxomatous mitral valve degeneration: 5HT and TGF-beta mechanisms. Cardiovasc Pathol 46:107196. doi:10.1016/j.carpath.2019.107196. 32006823 PMC7078050

[5] Pedersen HD, Haggstrom J. 2000. Mitral valve prolapse in the dog: a model of mitral valve prolapse in man. Cardiovasc Res 47:234–243. doi:10.1016/S0008-6363(00)00113-9. 10946060

[6] Karlin ET, Rush JE, Freeman LM. 2019. A pilot study investigating circulating trimethylamine *N*-oxide and its precursors in dogs with degenerative mitral valve disease with or without congestive heart failure. J Vet Intern Med 33:46–53. doi:10.1111/jvim.15347. 30511765 PMC6335534

[7] Li Q, Larouche-Lebel E, Loughran KA, Huh TP, Suchodolski JS, Oyama MA. 2021. Metabolomics analysis reveals deranged energy metabolism and amino acid metabolic reprogramming in dogs with myxomatous mitral valve disease. J Am Heart Assoc 10:e018923. doi:10.1161/JAHA.120.018923. 33890477 PMC8200728

[8] Wang Z, Klipfell E, Bennett BJ, Koeth R, Levison BS, Dugar B, Feldstein AE, Britt EB, Fu X, Chung YM, Wu Y, Schauer P, Smith JD, Allayee H, Tang WH, DiDonato JA, Lusis AJ, Hazen SL. 2011. Gut flora metabolism of phosphatidylcholine promotes cardiovascular disease. Nature 472:57–63. doi:10.1038/nature09922. 21475195 PMC3086762

[9] Tang WHW, Li DY, Hazen SL. 2019. Dietary metabolism, the gut microbiome, and heart failure. Nat Rev Cardiol 16:137–154. doi:10.1038/s41569-018-0108-7. 30410105 PMC6377322

[10] Witkowski M, Weeks TL, L HS. 2020. Gut microbiota and cardiovascular disease. Circ Res 127:553–570. doi:10.1161/CIRCRESAHA.120.316242. 32762536 PMC7416843

[11] Tang WH, Kitai T, Hazen SL. 2017. Gut microbiota in cardiovascular health and disease. Circ Res 120:1183–1196. doi:10.1161/CIRCRESAHA.117.309715. 28360349 PMC5390330

[12] Koeth RA, Wang Z, Levison BS, Buffa JA, Org E, Sheehy BT, Britt EB, Fu X, Wu Y, Li L, Smith JD, DiDonato JA, Chen J, Li H, Wu GD, Lewis JD, Warrier M, Brown JM, Krauss RM, Tang WH, Bushman FD, Lusis AJ, Hazen SL. 2013. Intestinal microbiota metabolism of l-carnitine, a nutrient in red meat, promotes atherosclerosis. Nat Med 19:576–585. doi:10.1038/nm.3145. 23563705 PMC3650111

[13] Lefebvre P, Cariou B, Lien F, Kuipers F, Staels B. 2009. Role of bile acids and bile acid receptors in metabolic regulation. Physiol Rev 89:147–191. doi:10.1152/physrev.00010.2008. 19126757

[14] Dawson PA, Lan T, Rao A. 2009. Bile acid transporters. J Lipid Res 50:2340–2357. doi:10.1194/jlr.R900012-JLR200. 19498215 PMC2781307

[15] Devlin AS, Fischbach MA. 2015. A biosynthetic pathway for a prominent class of microbiota-derived bile acids. Nat Chem Biol 11:685–690. doi:10.1038/nchembio.1864. 26192599 PMC4543561

[16] Ridlon JM, Kang DJ, Hylemon PB. 2006. Bile salt biotransformations by human intestinal bacteria. J Lipid Res 47:241–259. doi:10.1194/jlr.R500013-JLR200. 16299351

[17] Hylemon PB, Zhou H, Pandak WM, Ren S, Gil G, Dent P. 2009. Bile acids as regulatory molecules. J Lipid Res 50:1509–1520. doi:10.1194/jlr.R900007-JLR200. 19346331 PMC2724047

[18] Pluznick JL, Protzko RJ, Gevorgyan H, Peterlin Z, Sipos A, Han J, Brunet I, Wan LX, Rey F, Wang T, Firestein SJ, Yanagisawa M, Gordon JI, Eichmann A, Peti-Peterdi J, Caplan MJ. 2013. Olfactory receptor responding to gut microbiota-derived signals plays a role in renin secretion and blood pressure regulation. Proc Natl Acad Sci U S A 110:4410–4415. doi:10.1073/pnas.1215927110. 23401498 PMC3600440

[19] Marques FZ, Nelson E, Chu PY, Horlock D, Fiedler A, Ziemann M, Tan JK, Kuruppu S, Rajapakse NW, El-Osta A, Mackay CR, Kaye DM. 2017. High-fiber diet and acetate supplementation change the gut microbiota and prevent the development of hypertension and heart failure in hypertensive mice. Circulation 135:964–977. doi:10.1161/CIRCULATIONAHA.116.024545. 27927713

[20] Seo J, Matthewman L, Xia D, Wilshaw J, Chang YM, Connolly DJ. 2020. The gut microbiome in dogs with congestive heart failure: a pilot study. Sci Rep 10:13777. doi:10.1038/s41598-020-70826-0. 32792610 PMC7426839

[21] Kamo T, Akazawa H, Suda W, Saga-Kamo A, Shimizu Y, Yagi H, Liu Q, Nomura S, Naito AT, Takeda N, Harada M, Toko H, Kumagai H, Ikeda Y, Takimoto E, Suzuki JI, Honda K, Morita H, Hattori M, Komuro I. 2017. Dysbiosis and compositional alterations with aging in the gut microbiota of patients with heart failure. PLoS One 12:e0174099. doi:10.1371/journal.pone.0174099. 28328981 PMC5362204

[22] Katsimichas T, Ohtani T, Motooka D, Tsukamoto Y, Kioka H, Nakamoto K, Konishi S, Chimura M, Sengoku K, Miyawaki H, Sakaguchi T, Okumura R, Theofilis K, Iida T, Takeda K, Nakamura S, Sakata Y. 2018. Non-ischemic heart failure with reduced ejection fraction is associated with altered intestinal microbiota. Circ J 82:1640–1650. doi:10.1253/circj.CJ-17-1285. 29607983

[23] Cui X, Ye L, Li J, Jin L, Wang W, Li S, Bao M, Wu S, Li L, Geng B, Zhou X, Zhang J, Cai J. 2018. Metagenomic and metabolomic analyses unveil dysbiosis of gut microbiota in chronic heart failure patients. Sci Rep 8:635. doi:10.1038/s41598-017-18756-2. 29330424 PMC5766622

[24] Luedde M, Winkler T, Heinsen FA, Ruhlemann MC, Spehlmann ME, Bajrovic A, Lieb W, Franke A, Ott SJ, Frey N. 2017. Heart failure is associated with depletion of core intestinal microbiota. ESC Heart Fail 4:282–290. doi:10.1002/ehf2.12155. 28772054 PMC5542738

[25] Carrillo-Salinas FJ, Anastasiou M, Ngwenyama N, Kaur K, Tai A, Smolgovsky SA, Jetton D, Aronovitz M, Alcaide P. 2020. Gut dysbiosis induced by cardiac pressure overload enhances adverse cardiac remodeling in a T cell-dependent manner. Gut Microbes 12:1–20. doi:10.1080/19490976.2020.1823801. PMC7588211 33103561

[26] Katsimichas T, Antonopoulos AS, Katsimichas A, Ohtani T, Sakata Y, Tousoulis D. 2019. The intestinal microbiota and cardiovascular disease. Cardiovasc Res 115:1471–1486. doi:10.1093/cvr/cvz135. 31161201

[27] Kemis JH, Linke V, Barrett KL, Boehm FJ, Traeger LL, Keller MP, Rabaglia ME, Schueler KL, Stapleton DS, Gatti DM, Churchill GA, Amador-Noguez D, Russell JD, Yandell BS, Broman KW, Coon JJ, Attie AD, Rey FE. 2019. Genetic determinants of gut microbiota composition and bile acid profiles in mice. PLoS Genet 15:e1008073. doi:10.1371/journal.pgen.1008073. 31465442 PMC6715156

[28] Fung TC, Vuong HE, Luna CDG, Pronovost GN, Aleksandrova AA, Riley NG, Vavilina A, McGinn J, Rendon T, Forrest LR, Hsiao EY. 2019. Intestinal serotonin and fluoxetine exposure modulate bacterial colonization in the gut. Nat Microbiol 4:2064–2073. doi:10.1038/s41564-019-0540-4. 31477894 PMC6879823

[29] Yano JM, Yu K, Donaldson GP, Shastri GG, Ann P, Ma L, Nagler CR, Ismagilov RF, Mazmanian SK, Hsiao EY. 2015. Indigenous bacteria from the gut microbiota regulate host serotonin biosynthesis. Cell 161:264–276. doi:10.1016/j.cell.2015.02.047. 25860609 PMC4393509

[30] Oyama MA, Levy RJ. 2010. Insights into serotonin signaling mechanisms associated with canine degenerative mitral valve disease. J Vet Intern Med 24:27–36. doi:10.1111/j.1939-1676.2009.0411.x. 19912520

[31] Ljungvall I, Hoglund K, Lilliehook I, Oyama MA, Tidholm A, Tvedten H, Haggstrom J. 2013. Serum serotonin concentration is associated with severity of myxomatous mitral valve disease in dogs. J Vet Intern Med 27:1105–1112. doi:10.1111/jvim.12137. 23865457

[32] Arndt JW, Reynolds CA, Singletary GE, Connolly JM, Levy RJ, Oyama MA. 2009. Serum serotonin concentrations in dogs with degenerative mitral valve disease. J Vet Intern Med 23:1208–1213. doi:10.1111/j.1939-1676.2009.0378.x. 19709352

[33] Zhao C, Dong H, Zhang Y, Li Y. 2019. Discovery of potential genes contributing to the biosynthesis of short-chain fatty acids and lactate in gut microbiota from systematic investigation in *E. coli*. NPJ Biofilms Microbiomes 5:19. doi:10.1038/s41522-019-0092-7. 31312512 PMC6626047

[34] Chevrot R, Carlotti A, Sopena V, Marchand P, Rosenfeld E. 2008. *Megamonas rupellensis* sp. nov., an anaerobe isolated from the caecum of a duck. Int J Syst Evol Microbiol 58:2921–2924. doi:10.1099/ijs.0.2008/001297-0. 19060083

[35] Sakon H, Nagai F, Morotomi M, Tanaka R. 2008. *Sutterella parvirubra* sp. nov. and *Megamonas funiformis* sp. nov., isolated from human faeces. Int J Syst Evol Microbiol 58:970–975. doi:10.1099/ijs.0.65456-0. 18398204

[36] Reichardt N, Duncan SH, Young P, Belenguer A, McWilliam Leitch C, Scott KP, Flint HJ, Louis P. 2014. Phylogenetic distribution of three pathways for propionate production within the human gut microbiota. ISME J 8:1323–1335. doi:10.1038/ismej.2014.14. 24553467 PMC4030238

[37] Louis P, Flint HJ. 2017. Formation of propionate and butyrate by the human colonic microbiota. Environ Microbiol 19:29–41. doi:10.1111/1462-2920.13589. 27928878

[38] Chan YK, Brar MS, Kirjavainen PV, Chen Y, Peng J, Li D, Leung FC, El-Nezami H. 2016. High fat diet induced atherosclerosis is accompanied with low colonic bacterial diversity and altered abundances that correlates with plaque size, plasma A-FABP and cholesterol: a pilot study of high fat diet and its intervention with Lactobacillus rhamnosus GG (LGG) or telmisartan in ApoE^−/−^ mice. BMC Microbiol 16:264. doi:10.1186/s12866-016-0883-4. 27821063 PMC5100306

[39] Louis P, Young P, Holtrop G, Flint HJ. 2010. Diversity of human colonic butyrate-producing bacteria revealed by analysis of the butyryl-CoA:acetate CoA-transferase gene. Environ Microbiol 12:304–314. doi:10.1111/j.1462-2920.2009.02066.x. 19807780

[40] Eeckhaut V, Van Immerseel F, Croubels S, De Baere S, Haesebrouck F, Ducatelle R, Louis P, Vandamme P. 2011. Butyrate production in phylogenetically diverse Firmicutes isolated from the chicken caecum. Microb Biotechnol 4:503–512. doi:10.1111/j.1751-7915.2010.00244.x. 21375722 PMC3815262

[41] Gophna U, Konikoff T, Nielsen HB. 2017. Oscillospira and related bacteria - from metagenomic species to metabolic features. Environ Microbiol 19:835–841. doi:10.1111/1462-2920.13658. 28028921

[42] Segain JP, Raingeard de la Bletiere D, Bourreille A, Leray V, Gervois N, Rosales C, Ferrier L, Bonnet C, Blottiere HM, Galmiche JP. 2000. Butyrate inhibits inflammatory responses through NFkappaB inhibition: implications for Crohn's disease. Gut 47:397–403. doi:10.1136/gut.47.3.397. 10940278 PMC1728045

[43] McNeil NI, Cummings JH, James WP. 1978. Short chain fatty acid absorption by the human large intestine. Gut 19:819–822. doi:10.1136/gut.19.9.819. 30683 PMC1412179

[44] Rath S, Heidrich B, Pieper DH, Vital M. 2017. Uncovering the trimethylamine-producing bacteria of the human gut microbiota. Microbiome 5:54. doi:10.1186/s40168-017-0271-9. 28506279 PMC5433236

[45] AlShawaqfeh MK, Wajid B, Minamoto Y, Markel M, Lidbury JA, Steiner JM, Serpedin E, Suchodolski JS. 2017. A dysbiosis index to assess microbial changes in fecal samples of dogs with chronic inflammatory enteropathy. FEMS Microbiol Ecol 93:fix136. doi:10.1093/femsec/fix136. 29040443

[46] Guard BC, Honneffer JB, Jergens AE, Jonika MM, Toresson L, Lawrence YA, Webb CB, Hill S, Lidbury JA, Steiner JM, Suchodolski JS. 2019. Longitudinal assessment of microbial dysbiosis, fecal unconjugated bile acid concentrations, and disease activity in dogs with steroid-responsive chronic inflammatory enteropathy. J Vet Intern Med 33:1295–1305. doi:10.1111/jvim.15493. 30957301 PMC6524081

[47] Minamoto Y, Minamoto T, Isaiah A, Sattasathuchana P, Buono A, Rangachari VR, McNeely IH, Lidbury J, Steiner JM, Suchodolski JS. 2019. Fecal short-chain fatty acid concentrations and dysbiosis in dogs with chronic enteropathy. J Vet Intern Med 33:1608–1618. doi:10.1111/jvim.15520. 31099928 PMC6639498

[48] Giaretta PR, Rech RR, Guard BC, Blake AB, Blick AK, Steiner JM, Lidbury JA, Cook AK, Hanifeh M, Spillmann T, Kilpinen S, Syrja P, Suchodolski JS. 2018. Comparison of intestinal expression of the apical sodium-dependent bile acid transporter between dogs with and without chronic inflammatory enteropathy. J Vet Intern Med 32:1918–1926. doi:10.1111/jvim.15332. 30315593 PMC6271328

[49] Wahlstrom A, Sayin SI, Marschall HU, Backhed F. 2016. Intestinal crosstalk between bile acids and microbiota and its impact on host metabolism. Cell Metab 24:41–50. doi:10.1016/j.cmet.2016.05.005. 27320064

[50] Ridlon JM, Harris SC, Bhowmik S, Kang DJ, Hylemon PB. 2016. Consequences of bile salt biotransformations by intestinal bacteria. Gut Microbes 7:22–39. doi:10.1080/19490976.2015.1127483. 26939849 PMC4856454

[51] Sorg JA, Sonenshein AL. 2010. Inhibiting the initiation of *Clostridium difficile* spore germination using analogs of chenodeoxycholic acid, a bile acid. J Bacteriol 192:4983–4990. doi:10.1128/JB.00610-10. 20675492 PMC2944524

[52] Winston JA, Theriot CM. 2016. Impact of microbial derived secondary bile acids on colonization resistance against *Clostridium difficile* in the gastrointestinal tract. Anaerobe 41:44–50. doi:10.1016/j.anaerobe.2016.05.003. 27163871 PMC5050083

[53] Wang S, Martins R, Sullivan MC, Friedman ES, Misic AM, El-Fahmawi A, De Martinis ECP, O'Brien K, Chen Y, Bradley C, Zhang G, Berry ASF, Hunter CA, Baldassano RN, Rondeau MP, Beiting DP. 2019. Diet-induced remission in chronic enteropathy is associated with altered microbial community structure and synthesis of secondary bile acids. Microbiome 7:126. doi:10.1186/s40168-019-0740-4. 31472697 PMC6717631

[54] Wang H, Latorre JD, Bansal M, Abraha M, Al-Rubaye B, Tellez-Isaias G, Hargis B, Sun X. 2019. Microbial metabolite deoxycholic acid controls *Clostridium perfringens-*induced chicken necrotic enteritis through attenuating inflammatory cyclooxygenase signaling. Sci Rep 9:14541. doi:10.1038/s41598-019-51104-0. 31601882 PMC6787040

[55] Mayerhofer CCK, Ueland T, Broch K, Vincent RP, Cross GF, Dahl CP, Aukrust P, Gullestad L, Hov JR, Troseid M. 2017. Increased secondary/primary bile acid ratio in chronic heart failure. J Card Fail 23:666–671. doi:10.1016/j.cardfail.2017.06.007. 28688889

[56] Kitahara M, Takamine F, Imamura T, Benno Y. 2001. *Clostridium hiranonis* sp. nov., a human intestinal bacterium with bile acid 7alpha-dehydroxylating activity. Int J Syst Evol Microbiol 51:39–44. doi:10.1099/00207713-51-1-39. 11211270

[57] Adams SH, Hoppel CL, Lok KH, Zhao L, Wong SW, Minkler PE, Hwang DH, Newman JW, Garvey WT. 2009. Plasma acylcarnitine profiles suggest incomplete long-chain fatty acid beta-oxidation and altered tricarboxylic acid cycle activity in type 2 diabetic African-American women. J Nutr 139:1073–1081. doi:10.3945/jn.108.103754. 19369366 PMC2714383

[58] Spiekerkoetter U, Sun B, Zytkovicz T, Wanders R, Strauss AW, Wendel U. 2003. MS/MS-based newborn and family screening detects asymptomatic patients with very-long-chain acyl-CoA dehydrogenase deficiency. J Pediatr 143:335–342. doi:10.1067/S0022-3476(03)00292-0. 14517516

[59] Shekhawat PS, Matern D, Strauss AW. 2005. Fetal fatty acid oxidation disorders, their effect on maternal health and neonatal outcome: impact of expanded newborn screening on their diagnosis and management. Pediatr Res 57:78R–86R. doi:10.1203/01.PDR.0000159631.63843.3E. PMC3582391 15817498

[60] Cheng ML, Wang CH, Shiao MS, Liu MH, Huang YY, Huang CY, Mao CT, Lin JF, Ho HY, Yang NI. 2015. Metabolic disturbances identified in plasma are associated with outcomes in patients with heart failure: diagnostic and prognostic value of metabolomics. J Am Coll Cardiol 65:1509–1520. doi:10.1016/j.jacc.2015.02.018. 25881932

[61] Hunter WG, Kelly JP, McGarrah RW, III, Khouri MG, Craig D, Haynes C, Ilkayeva O, Stevens RD, Bain JR, Muehlbauer MJ, Newgard CB, Felker GM, Hernandez AF, Velazquez EJ, Kraus WE, Shah SH. 2016. Metabolomic profiling identifies novel circulating biomarkers of mitochondrial dysfunction differentially elevated in heart failure with preserved versus reduced ejection fraction: evidence for shared metabolic impairments in clinical heart failure. J Am Heart Assoc 5:e003190. doi:10.1161/JAHA.115.003190. 27473038 PMC5015273

[62] Li Q, Heaney A, Langenfeld-McCoy N, Boler BV, Laflamme DP. 2019. Dietary intervention reduces left atrial enlargement in dogs with early preclinical myxomatous mitral valve disease: a blinded randomized controlled study in 36 dogs. BMC Vet Res 15:425. doi:10.1186/s12917-019-2169-1. 31775756 PMC6882217

[63] Li Q, Laflamme DP, Bauer JE. 2020. Serum untargeted metabolomic changes in response to diet intervention in dogs with preclinical myxomatous mitral valve disease. PLoS One 15:e0234404. doi:10.1371/journal.pone.0234404. 32555688 PMC7302913

[64] Cornell CC, Kittleson MD, Della Torre P, Haggstrom J, Lombard CW, Pedersen HD, Vollmar A, Wey A. 2004. Allometric scaling of M-mode cardiac measurements in normal adult dogs. J Vet Intern Med 18:311–321. doi:10.1111/j.1939-1676.2004.tb02551.x. 15188817

[65] Li Q, Pan Y. 2020. Differential responses to dietary protein and carbohydrate ratio on gut microbiome in obese vs. lean cats. Front Microbiol 11:591462. doi:10.3389/fmicb.2020.591462. 33178173 PMC7596662

[66] Moreau NM, Goupry SM, Antignac JP, Monteau FJ, Le Bizec BJ, Champ MM, Martin LJ, Dumon HJ. 2003. Simultaneous measurement of plasma concentrations and ^13^C-enrichment of short-chain fatty acids, lactic acid and ketone bodies by gas chromatography coupled to mass spectrometry. J Chromatogr B Analyt Technol Biomed Life Sci 784:395–403. doi:10.1016/s1570-0232(02)00827-9. 12505787

[67] Oksanen J, Blanchet FG, Friendly M, Kindt R, Legendre P, McGlinn D, Minchin PR, O'Hara RB, Simpson GL, Solymos P, Stevens MHH, Szoecs E, Wagner H. 2019. vegan: community ecology package. R package version 25-6.

[68] Morgan XC, Tickle TL, Sokol H, Gevers D, Devaney KL, Ward DV, Reyes JA, Shah SA, LeLeiko N, Snapper SB, Bousvaros A, Korzenik J, Sands BE, Xavier RJ, Huttenhower C. 2012. Dysfunction of the intestinal microbiome in inflammatory bowel disease and treatment. Genome Biol 13:R79. doi:10.1186/gb-2012-13-9-r79. 23013615 PMC3506950

