# Supplementary figures and images for: Gut Dysbiosis and Its Associations with Gut Microbiota-Derived Metabolites in Dogs with Myxomatous Mitral Valve Disease
Source: mSystems. 2021 Apr 20;6(2):e00111-21. doi: 10.1128/mSystems.00111-21 (PMC8546968; doi:10.1128/mSystems.00111-21)

A

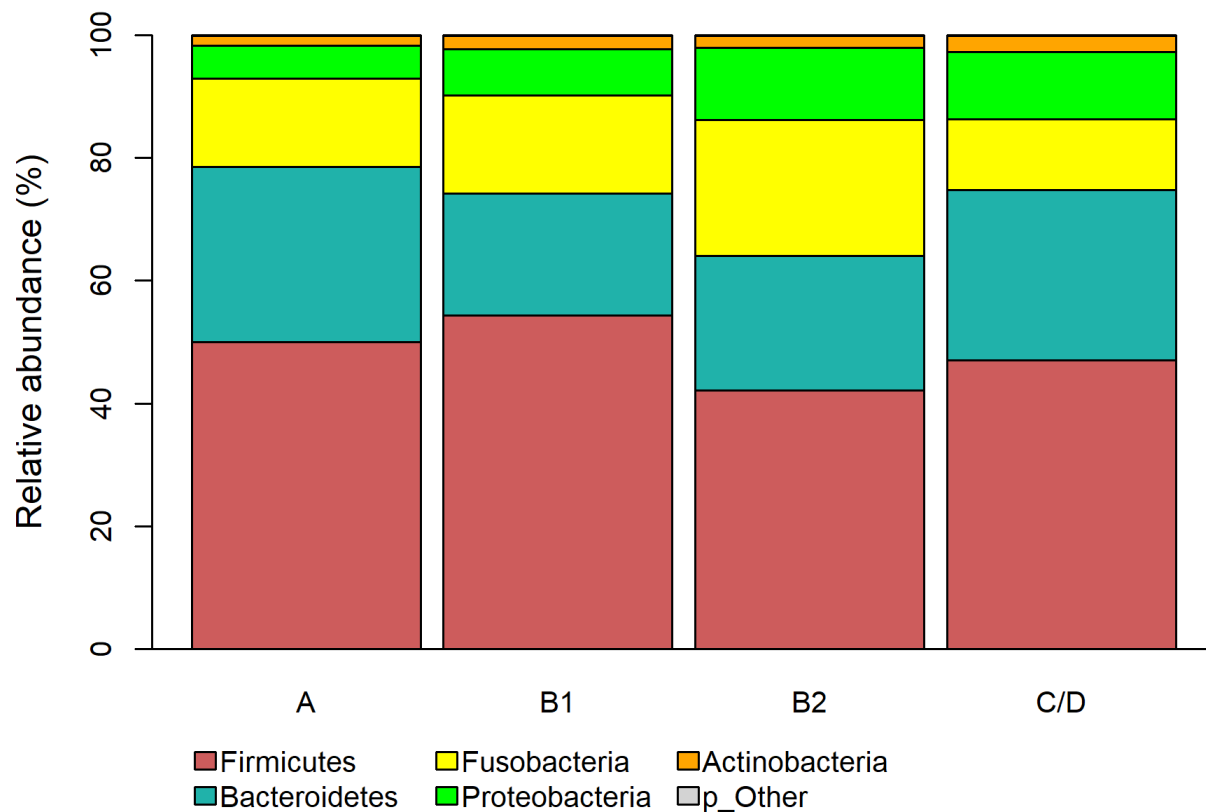

B

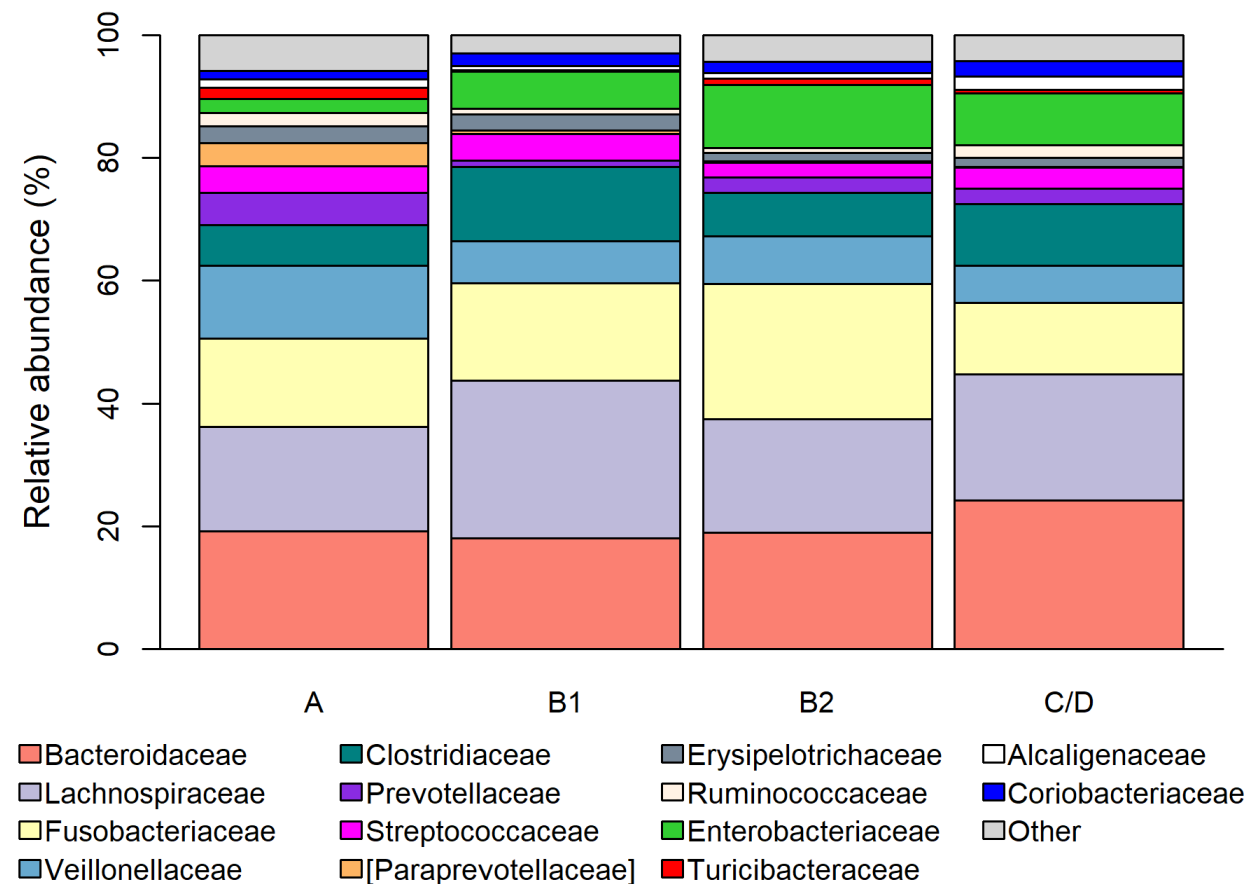

Supplement: FIG S1 [file msystems.00111-21-sf001.pdf]

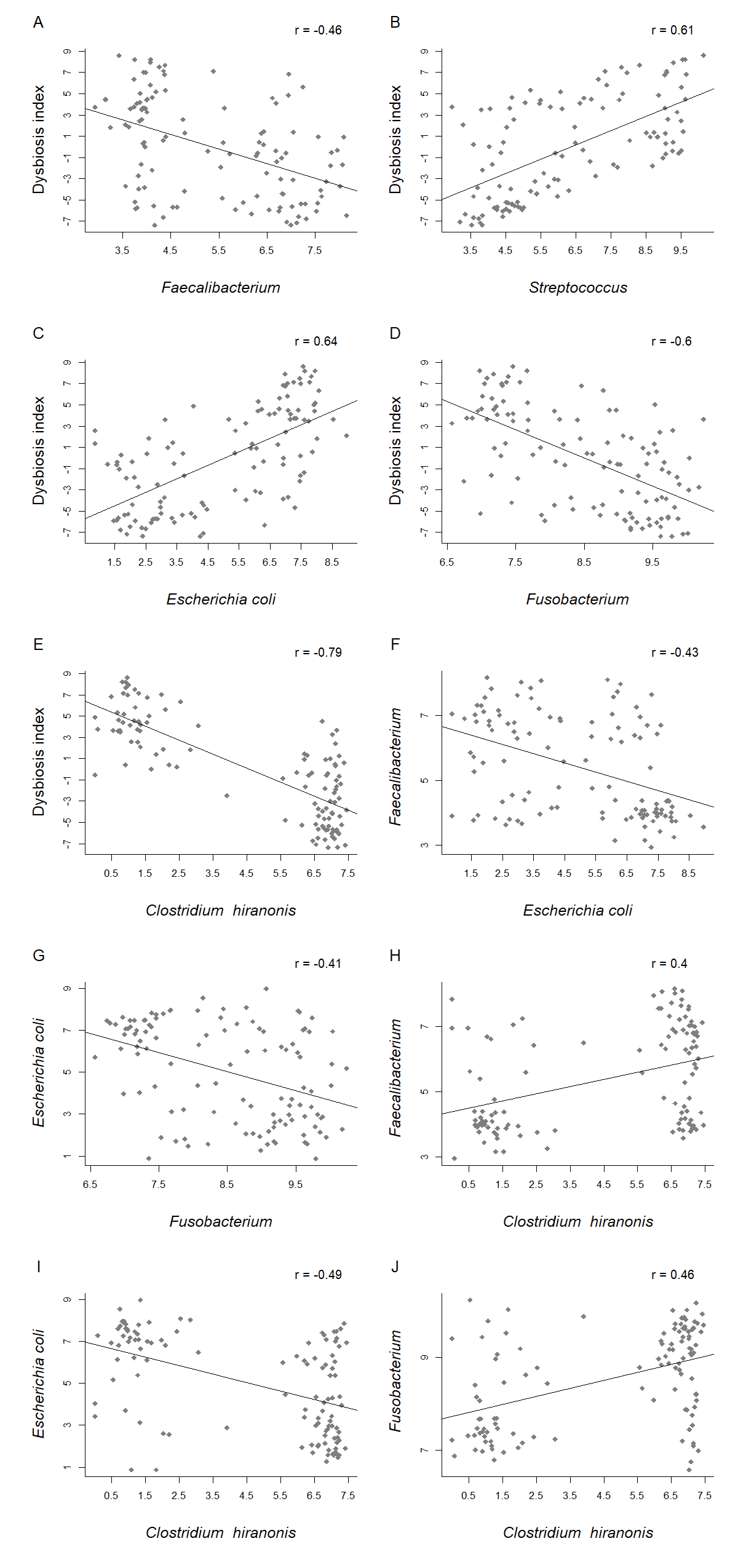

Supplement: FIG S2 [file msystems.00111-21-sf002.tiff]
