# Supplementary material for: Gut Dysbiosis and Its Associations with Gut Microbiota-Derived Metabolites in Dogs with Myxomatous Mitral Valve Disease
Source: mSystems. 2021 Apr 20;6(2):e00111-21. doi: 10.1128/mSystems.00111-21 (PMC8546968; doi:10.1128/mSystems.00111-21)
Supplement: TEXT S1 [file msystems.00111-21-s0001.docx]

1. **METHOD SUMMARY**

The Human Feces Bile Acid Panel measures all of the major primary and secondary bile acids and their conjugates: Cholic Acid (CA), Chenodeoxycholic Acid (CDCA), Deoxycholic Acid (DCA), Lithocholic Acid (LCA), Ursodeoxycholic Acid (UDCA), Glycocholic Acid (GCA), Glycochenodeoxycholic Acid (GCDCA), Glycodeoxycholic Acid (GDCA), Glycoursodeoxycholic Acid (GUDCA), Taurocholic Acid (TCA), Taurochenodeoxycholic Acid (TCDCA), Taurodeoxycholic Acid (TDCA), Taurolithocholic Acid (TLCA), Tauroursodeoxycholic Acid (TUDCA), and Glycolithocholic Acid (GLCA). Bile acid concentrations are analyzed by LC- MS/MS (Metabolon Method TAM178: “LC-MS/MS Method for the Quantitation of Bile Acids”).

Calibration samples are prepared at eight different concentration levels by spiking an acidified methanol solution with corresponding calibration spiking solutions. Calibration samples, study samples, and quality control samples are spiked with a solution of labeled internal standards and subjected to protein precipitation with an organic solvent (acidified methanol). Following centrifugation, an aliquot of the organic supernatant is evaporated to dryness in a gentle stream of nitrogen. The dried extracts are reconstituted and injected onto an Agilent 1290 Infinity / Sciex QTRAP 6500 LC-MS/MS system equipped with a C18 reverse phase UHPLC column. The mass spectrometer is operated in negative mode using electrospray ionization (ESI).

The peak area of each bile acid parent (pseudo-MRM mode) or product ion is measured against the peak area of the respective internal standard parent (pseudo-MRM mode) or product ion. Quantitation is performed using a weighted linear least squares regression analysis generated from fortified calibration standards prepared immediately prior to each run.

LC-MS/MS raw data are collected using AB SCIEX software Analyst 1.6.2 and processed using SCIEX OS-MQ software v1.7. Data reduction is performed using Microsoft Excel for Office 365 v.16.

1. **SAMPLE ANALYSIS**

Dog feces samples were analyzed for 15 bile acids by LC-MS/MS according to Metabolon’s protocol.

Four levels of QC were prepared. Two of the levels (QC Low and QC High) were prepared in a surrogate matrix by spiking with stock solutions to obtain the appropriate concentrations for each level. The other two levels (QC Medium and QC ALOQ) were prepared in human feces either at endogenous levels or by spiking with stock solutions to obtain the appropriate concentrations for each level.

Sample analysis was carried out in a 96-well plate format containing two calibration curves and eight QC samples per plate to monitor assay performance. Two batches were prepared and analyzed undiluted and at a 100-fold dilution.

Accuracy was evaluated using the corresponding QC replicates in the sample runs. (QC acceptance criteria: at least 50% of QC samples at each concentration level per analyte should be within

±20.0% of the set mean, and at least 2/3 of all QC samples per analyte should fall within ±20.0% of the corresponding set mean.). QCs met acceptance criteria at all levels for all analytes. Detailed results are presented in Table 2.

Analyte concentrations that fell below the limit of quantitation are reported as BLOQ instead of a

value. Samples that had analyte concentrations that quantitated above the limit of quantitation were subsequently analyzed at a 100-fold dilution. Concentration data for the ALOQ analytes are reported from the diluted samples after taking the dilution factor into account. There were twelve (12) samples with analyte concentrations that quantitated above the limit of quantitation even with

a 100-fold dilution. These concentrations were extrapolated, and the reported values given a comment of ALOQ.
