# Supplementary material for: Gut Dysbiosis and Its Associations with Gut Microbiota-Derived Metabolites in Dogs with Myxomatous Mitral Valve Disease
Source: mSystems. 2021 Apr 20;6(2):e00111-21. doi: 10.1128/mSystems.00111-21 (PMC8546968; doi:10.1128/mSystems.00111-21)
Supplement: TABLE S2 [file msystems.00111-21-st002.docx]

**Table S2. QC Accuracy for Bile Acids**

| Analyte Name | QC Low | | QC Medium | | QC High | | QC ALOQ | |
| --- | --- | --- | --- | --- | --- | --- | --- | --- |
|  | Average Concentration (ng/mg) | Average Accuracy (%) | Average Concentration (ng/mg) | Average Accuracy (%) | Average Concentration (ng/mg) | Average Accuracy (%) | Average Concentration (ng/mg) | Average Accuracy (%) |
| Chenodeoxycholic Acid | 1.50 | 100 | 40.6 | 94.5 | 188 | 101 | 1371 | 94.2 |
| Cholic Acid | 0.813 | 108 | 18.6 | 101 | 93.4 | 102 | 673 | 102 |
| Deoxycholic Acid | 1.58 | 105 | 53.7 | 94.0 | 189 | 101 | 2566 | 98.6 |
| Glycochenodeoxycholic Acid | 1.45 | 96.7 | 30.3 | 98.2 | 188 | 101 | 1238 | 104 |
| Glycocholic Acid | 0.740 | 98.7 | 14.5 | 98.6 | 93.6 | 102 | 639 | 100 |
| Glycodeoxycholic Acid | 0.746 | 99.4 | 16.3 | 103 | 98.1 | 104 | 649 | 106 |
| Glycolithocholic Acid | 0.744 | 99.2 | 16.3 | 102 | 94.8 | 102 | 610 | 99.7 |
| Glycoursodeoxycholic Acid | 1.46 | 97.4 | 32.0 | 107 | 192 | 104 | 1274 | 96.9 |
| Lithocholic Acid | 0.731 | 97.4 | 25.3 | 93.8 | 94.2 | 103 | 740 | 101 |
| Taurochenodeoxycholic Acid | 1.46 | 97.3 | 37.5 | 100 | 179 | 96.3 | 1221 | 99.6 |
| Taurocholic Acid | 0.739 | 98.5 | 16.6 | 107 | 96.1 | 104 | 611 | 104 |
| Taurodeoxycholic Acid | 1.53 | 102 | 35.1 | 109 | 184 | 99.5 | 1246 | 110 |
| Taurolithocholic Acid | 0.757 | 101 | 20.2 | 107 | 94.3 | 101 | 630 | 99.7 |
| Tauroursodeoxycholic Acid | 0.740 | 98.7 | 17.2 | 106 | 94.5 | 102 | 657 | 104 |
| Ursodeoxycholic Acid | 1.48 | 98.6 | 33.9 | 96.9 | 189 | 102 | 1188 | 89.8 |

**GLOSSARY OF TERMS**

| ALQ or ALOQ | Above the Limit of Quantitation |
| --- | --- |
| BLQ or BLOQ | Below the Limit of Quantitation |
| UHPLC | Ultra High Performance Liquid Chromatography |
| LLOQ or LLQ | Lower Limit of Quantitation |
| QC | Quality Control |
| ULOQ | Upper Limit of Quantitation |
